# VDR restores the expression of PINK1 and BNIP3 in TECs of streptozotocin-induced diabetic mice

**DOI:** 10.26508/lsa.202302474

**Published:** 2024-05-02

**Authors:** Cheng Yang, Bin Yi, Shikun Yang, Aimei Li, Jishi Liu, Jianwen Wang, Jun Liu, Zhi Li, Qin Liao, Wei Zhang, Hao Zhang

**Affiliations:** 1 Department of Nephrology, The Third Xiangya Hospital , Central South University, Changsha, China; 2 Clinical Research Center for Critical Kidney Disease in Hunan Province, Changsha, China; 3 Department of Anesthesiology, The Third Xiangya Hospital , Central South University, Changsha, China

## Abstract

Defective mitophagy in renal tubular epithelial cells is one of the main drivers of renal fibrosis in diabetic kidney disease. Our gene sequencing data showed the expression of PINK1 and BNIP3, two key molecules of mitophagy, was decreased in renal tissues of VDR-knockout mice. Herein, streptozotocin (STZ) was used to induce renal interstitial fibrosis in mice. VDR deficiency exacerbated STZ-induced renal impairment and defective mitophagy. Paricalcitol (pari, a VDR agonist) and the tubular epithelial cell–specific overexpression of VDR restored the expression of PINK1 and BNIP3 in the renal cortex and attenuated STZ-induced kidney fibrosis and mitochondrial dysfunction. In HK-2 cells under high glucose conditions, an increased level of α-SMA, COL1, and FN and a decreased expression of PINK1 and BNIP3 with severe mitochondrial damage were observed, and these alterations could be largely reversed by pari treatment. ChIP-qPCR and luciferase reporter assays showed VDR could positively regulate the transcription of *Pink1* and *Bnip3* genes. These findings reveal that VDR could restore mitophagy defects and attenuate STZ-induced fibrosis in diabetic mice through regulation of PINK1 and BNIP3.

## Abstract (precis)

VDR protects against fibrosis and defective mitophagy in diabetic mice by regulating mitophagy-related proteins PINK1 and BNIP3.

## Graphical Abstract

**Figure:**
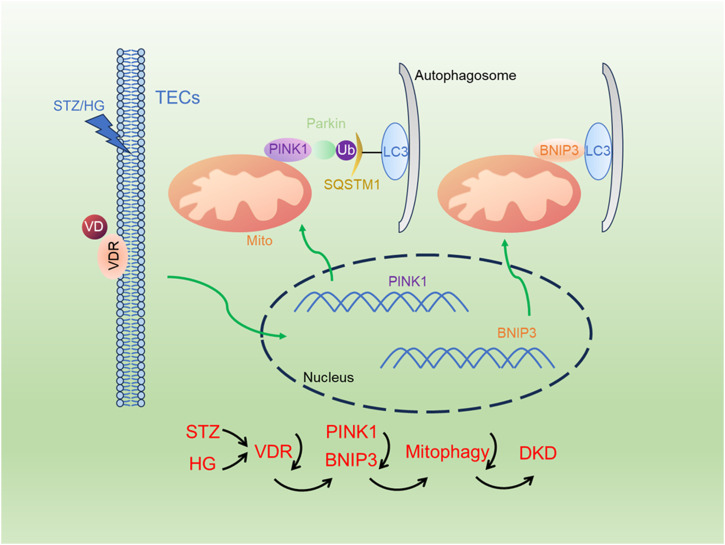

## Introduction

Over 500 million adults worldwide are living with diabetes (1), and at least one-third of them will develop diabetic kidney disease (DKD) or even end-stage kidney disease (2, 3). Although glucagon-like peptide-1 receptor agonists, sodium–glucose cotransporter-2 inhibitors, and other antidiabetic drugs showed beneficial effects on diabetic patients (4), it is imperative to seek new effective drugs to treat DKD.

Renal fibrosis is the key pathophysiological hallmark in the progression of DKD to end-stage kidney disease (5, 6). It has been accepted that proximal tubule injury occurs before glomerular lesions in early diabetic nephropathy (DN) in recent years and can further mediate glomerular injury (7). Therefore, inhibiting tubular epithelial cell (TEC) fibrosis could be a promising strategy for preventing DKD progression.

Mitochondria-specific autophagy, also known as mitophagy, is an autophagic process that selectively clears damaged mitochondria to maintain mitochondrial homeostasis (8, 9, 10). Among renal resident cells, TECs have the highest mitochondrial content and ATP consumption to drive the transport of glucose, ions, and other nutrients (8). As a result, mitochondrial dysfunction in TECs may cause serious renal damage. Impaired mitophagy could not only induce abnormal lipid deposition and glucose metabolism but also exacerbate renal fibrosis (11, 12). PTEN-induced putative kinase 1 (PINK1) and BCL2 interacting protein 3 (BNIP3) are two of the key proteins that mediate mitophagy pathways by binding with microtubule-associated protein light chain 3 (LC3), a factor mostly used to detect autophagy (13, 14). Studies have demonstrated that both PINK1 and BNIP3-dependent mitophagy are involved in DKD (15, 16). However, the regulatory mechanisms of mitophagy in TECs or in DKD remain largely unclear.

Vitamin D receptor (VDR), which is sensitive to its ligand 1,25-dihydroxyvitamin D3 or other agonists, belongs to the nuclear receptor superfamily and could enter the nucleus to regulate the transcription of its target genes (17, 18). VDR is involved in the pathological mechanism of multiple kidney diseases, including DKD, through immune regulation and anti-fibrosis (19, 20, 21). Previously, we observed that VDR expression was down-regulated in peripheral blood mononuclear cells and TECs from patients with DN (22). Our recent study reported that VDR works as an anti-inflammatory factor in DKD by ameliorating autophagy disorders via the AMPK pathway in streptozotocin (STZ)-induced diabetic mice (23). However, the effects of VDR on mitophagy and fibrosis in diabetic mice and HK-2 cells under high glucose (HG) conditions were not clarified. Here, we used two genetically modified mouse models with VDR-knockout (KO) and TEC-specific VDR overexpression (VDR-OE) to clarify the effects of VDR in repairing mitophagy and reducing fibrosis in DKD, as well as the underlying mechanism.

## Results

### VDR deficiency down-regulates the mitophagy-related proteins PINK1 and BNIP3 in C57 mice

**Figure 1.**
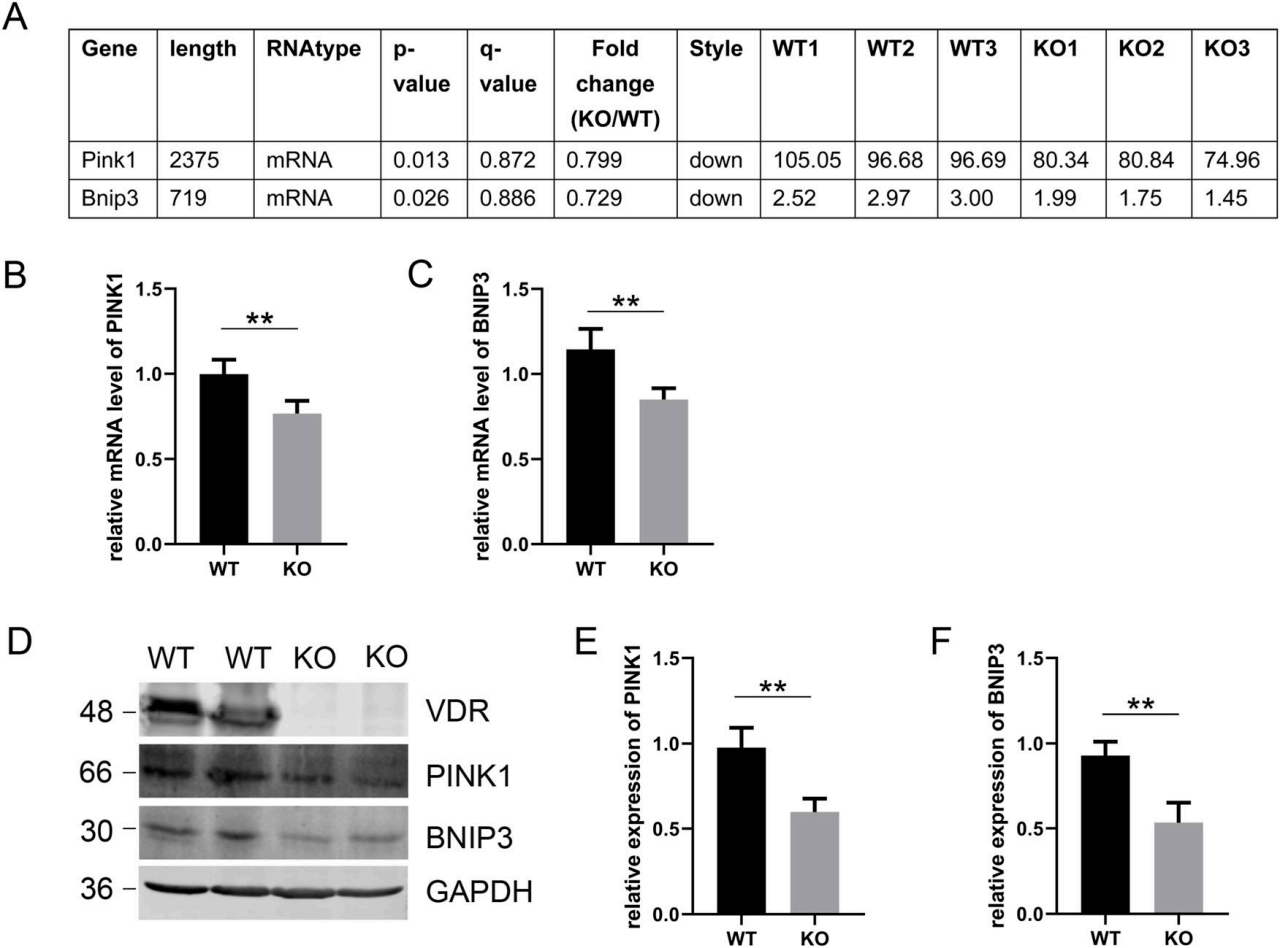
VDR loss leads to down-regulation of BNIP3 and PINK1 in C57 mice. **(A)** Down-regulated mRNA expression of Bnip3 and Pink1 from the whole RNA-sequencing and analysis. **(B, C)** mRNA levels of PINK1 (B) and BNIP3(C) in the cortex of WT and KO mice. **(D)** Representative blots of VDR, PINK1, and BNIP3 in the cortex of WT and KO mice. GAPDH was used as a loading control. **(E, F)** Densitometric analysis of PINK1 and BNIP3 between the WT and KO group. The data in (A, B, C, E, F) are presented as the mean ± SD. A two-tailed unpaired *t* test was used. *P* < 0.05 was considered to be statistically significant. **P < 0.01. n ≥ 5. Source data are available for this figure.

To explore the renoprotective effect of VDR, we first constructed *Vdr*^+/+^ and *Vdr*^−/−^ C57BL/6 mouse models as previously described (17, 23). We performed a whole RNA-sequencing analysis of renal cortex tissues from VDR-KO mice. The results showed that the mRNA levels of BNIP3 and PINK1 in KO mice were lower than those in WT mice (Fig 1A). Subsequent validation by real-time quantitative PCR (qPCR) and Western blot analysis showed consistent decreases of PINK1 and BNIP3 in the renal cortex of VDR-KO mice compared with WT mice (Fig 1B–F). Because PINK1 and BNIP3 are critical regulators of mitophagy (24), these data indicated a potential regulation of VDR in mitophagy.

### Lack of VDR exacerbates diabetic kidney injury and fibrosis in STZ mice

**Figure 2.**
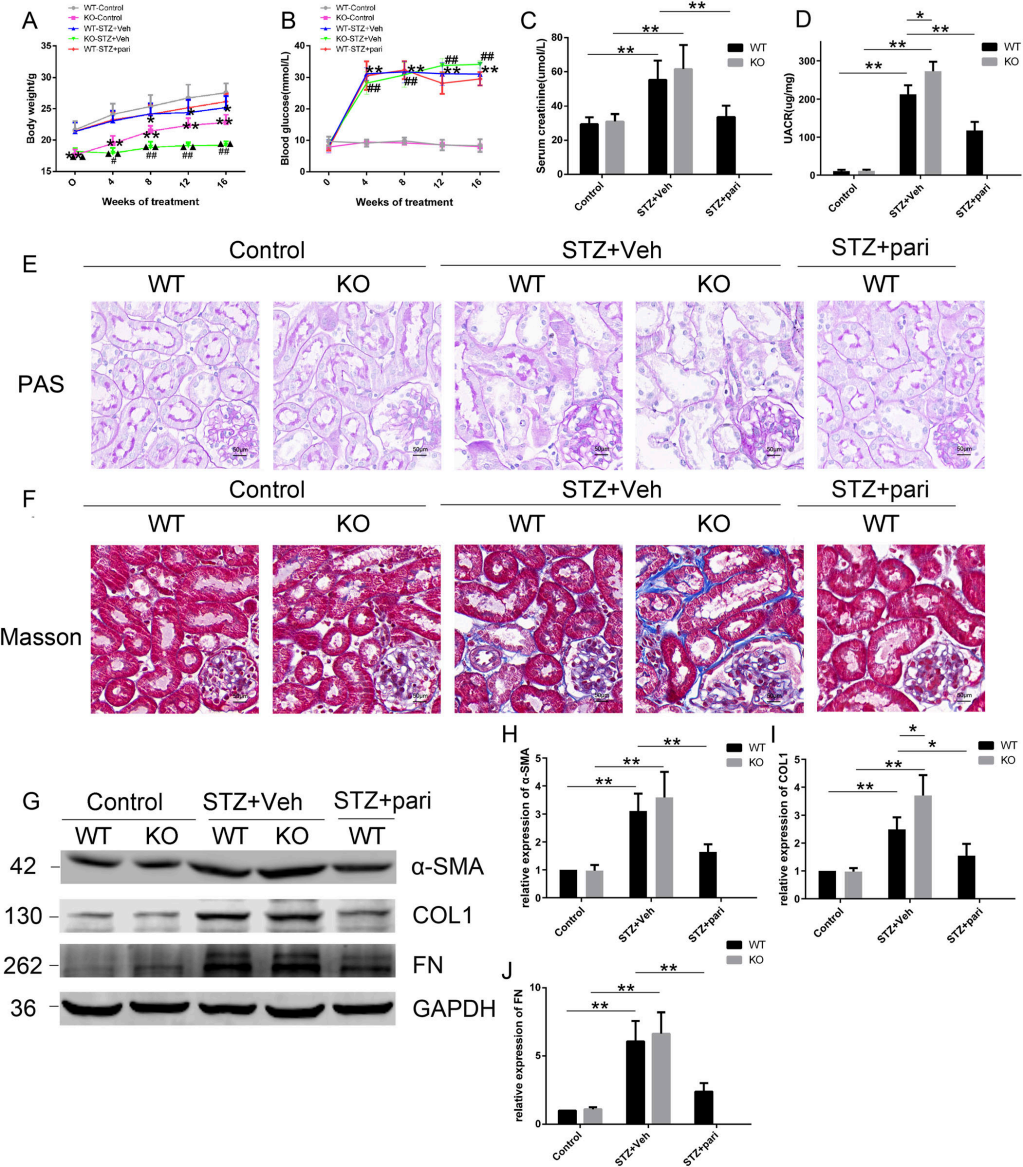
VDR deficiency reinforces kidney fibrosis in STZ-induced diabetic mice. **(A)** Changes in body weight after STZ injections every 4 wk. **(B)** Blood glucose fluctuation was measured every 4 wk within 16 wk after STZ injections. The data in (A, B) are presented as the mean ± SD, *P < 0.05, **P < 0.01 compared with the WT-control group, ^#^*P* < 0.05, ^##^*P* < 0.01 compared with the KO-control group, and ^▲▲^*P* < 0.01 compared with the WT-STZ group. **(C)** Serum creatinine levels at 16 wk after STZ injections. **(D)** Urinary ACR values at 16 wk after STZ injections. **(E)** Representative images of PAS staining. **(F)** Representative images of the Masson staining at 16 wk after STZ injections. Scale bar = 50 μm. **(G, H, I, J)** Representative blots and densitometric analysis of fibrosis markers (α-SMA, COL1, and FN) in the kidney of the indicated groups at 16 wk after STZ injections or vehicle. GAPDH was set as a loading control. The bands of proteins were measured with ImageJ software. n = 5 mice. The data are presented as the mean ± SD, *P < 0.05, **P < 0.01. Source data are available for this figure.

To clarify whether VDR regulates mitophagy in diabetes-induced renal fibrosis, we established STZ-induced diabetic mouse model. We monitored body weight, blood glucose, serum creatinine, and the urinary albumin-to-creatinine ratio (UACR) in STZ-induced diabetic mice with or without VDR-KO or paricalcitol (pari) treatment. VDR-KO mice exhibited the worst mobility after 16 wk of STZ injection among the five groups. All diabetic mice showed slight weight loss in comparison with nondiabetic mice. There was little difference in body weight between the WT-STZ group and the WT-STZ+pari group, suggesting that paricalcitol treatment did not influence mouse body weight (Fig 2A). In addition, we monitored blood glucose levels every 4 wk. The blood glucose levels of STZ-induced mice were significantly increased over time, and the administration of paricalcitol had no effect on blood glucose levels of the diabetic mice (Fig 2B). The mice in the KO-STZ group had higher serum creatinine levels than those in the WT-STZ group. Moreover, paricalcitol treatment (WT-STZ+pari group) effectively abrogated the increase in serum creatinine in diabetic mice (WT-STZ group) (Fig 2C). Among the five groups, the KO-STZ group had the most significant increase in the UACR, and paricalcitol injection reduced the UACR in the WT-STZ group (Fig 2D).

To detect the effects of VDR loss on histological injury in mice, we examined renal lesions by periodic acid–Schiff (PAS) staining after 16 wk of STZ injections. WT diabetic mice had glomerular enlargement and mesangial cell destruction, including disruption of the brush border or TECs and obvious renal tubular injury, whereas VDR-KO resulted in more severe renal tubular injury in STZ-induced mice than that in the WT-STZ group. In contrast, VDR agonist paricalcitol markedly attenuated tubular abnormalities induced by STZ in WT mice (Fig 2E).

**Figure S1.**
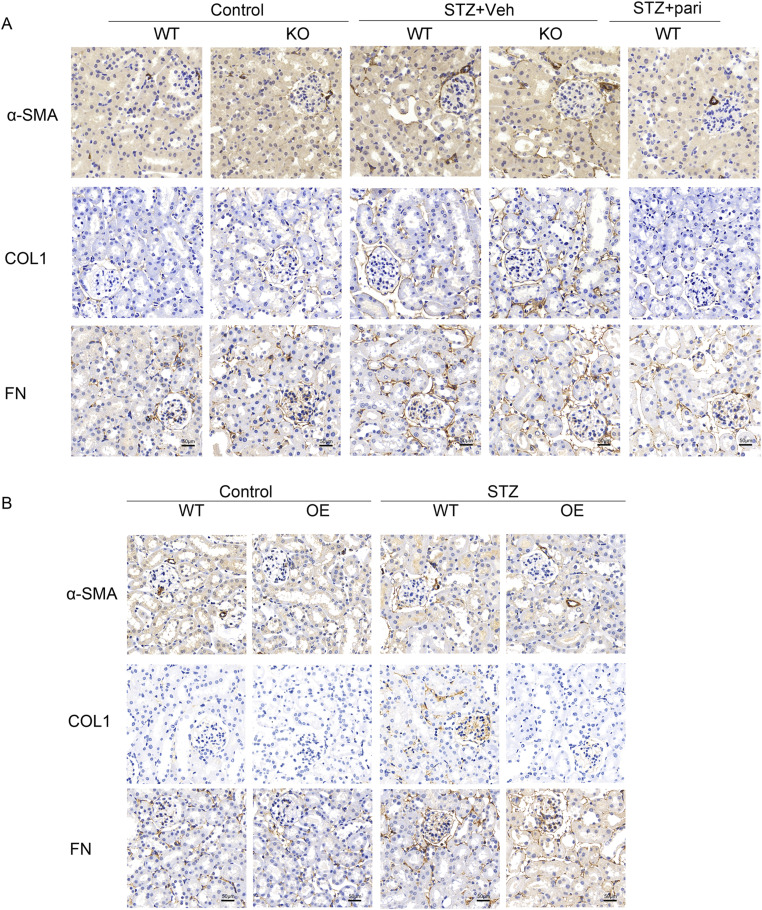
Loss of VDR exacerbates renal fibrosis in STZ mice, and the VDR agonist or VDR overexpression could alleviate renal fibrosis. **(A, B)** Representative images of IHC staining of α-SMA, COL1, and FN in the kidney at 16 wk after STZ injections. Scale bar = 50 μm.

After 16 wk of STZ injections, we performed the Masson staining of renal tissues and detected the expression of alpha-smooth muscle actin (α-SMA), type I collagen (COL1), and fibronectin (FN) by Western blotting, which are typical indicators of fibrosis. Masson’s trichrome staining showed that STZ significantly induced tubulointerstitial fibrosis in WT mice, and a much more pronounced fibrotic phenotype was observed in the KO-STZ group. On the contrary, paricalcitol treatment reduced collagen fiber deposition induced by STZ in WT mice (Fig 2F). Western blot in Fig 2G–J and immunohistochemical (IHC) staining in Fig S1A show that the α-SMA, COL1, and FN levels of diabetic mice were up-regulated compared with controls, and the expression of these fibrosis molecules was more obviously increased in the KO-STZ group than in the WT-STZ group. Paricalcitol treatment could effectively inhibit the expression of these fibrosis molecules in diabetes. These data indicate that VDR deficiency could accentuate renal injury and fibrosis in STZ-induced diabetic mice.

### Loss of VDR exacerbates mitophagy dysfunction in STZ-induced mice

**Figure 3.**
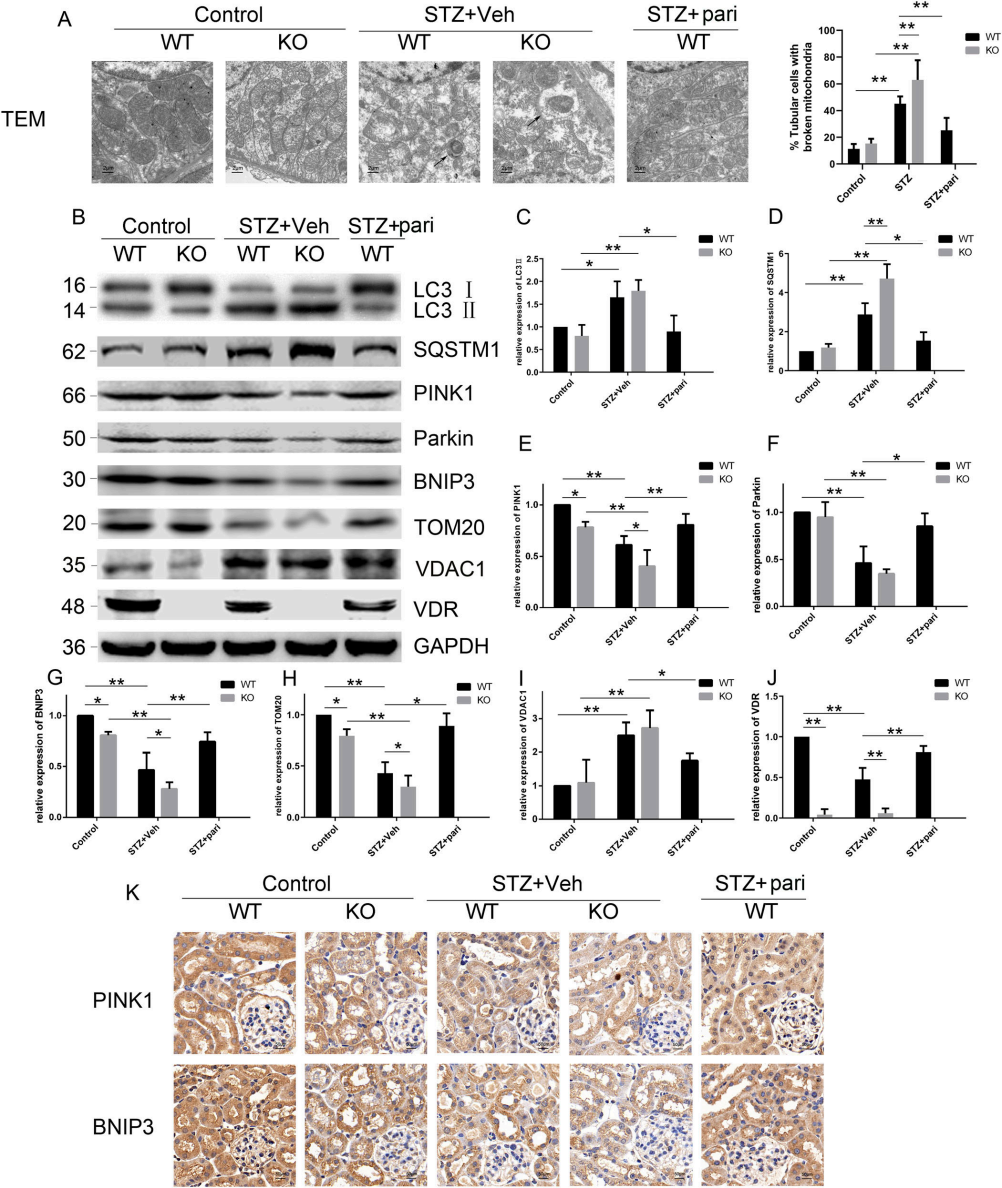
Loss of VDR aggravates mitophagy defects in STZ mice. **(A)** Representative images of TEM (left) showing the structure of mitochondria in TECs of mice among five groups at 12 wk after STZ injections and quantification of broken mitochondria (right). Black arrows indicate the autophagosomes, which wrap the damaged mitochondria. Scale bars = 2 μm. **(B)** Representative blots of LC3, SQSTM1, PINK1, Parkin, BNIP3, TOM20, VDAC1, and VDR. GAPDH was set as a loading control. **(C, D, E, F, G, H, I, J)** Densitometric analysis of LC3, SQSTM1, PINK1, Parkin, BNIP3, TOM20, VDAC1, and VDR in the indicated groups. Values are the mean ± SEM, *P < 0.05, **P < 0.01 (two-way ANOVA and one-way ANOVA). **(K)** Representative images of IHC staining in the renal cortex of the indicated groups at 12 wk after STZ injections. Scale bar = 50 μm. n = 5. Source data are available for this figure.

To explore the role of VDR in mitophagy in DKD, we examined mitochondrial alterations and mitophagy status in diabetic contexts. We used transmission electron microscopy (TEM) to observe the mitochondrial structure of TECs in mice. The results showed that the morphological structure of mitochondria in the TECs of diabetic mice was abnormal and characterized by mitochondrial swelling and increased fragmentation. In the WT-STZ group, a large number of mitochondrial cristae were broken, and mitochondrial matrix particles were missing (Fig 3A). Moreover, in the KO-STZ group, more mitochondrial crest had disappeared, and more mitochondrial membranes were incomplete. These mitochondrial abnormalities could be largely restored by paricalcitol treatment in WT mice (Fig 3A).

**Figure S2.**
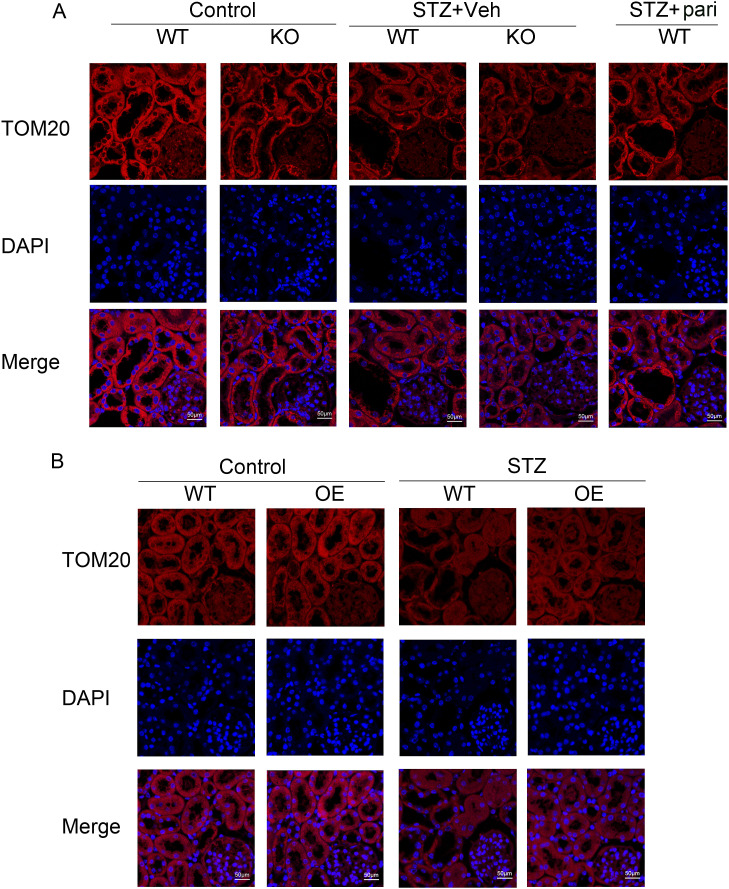
VDR deficiency aggravated the decrease of TOM20 expression in STZ mice, and TOM20 expression could be restored in VDR-OE STZ mice or by pari treatment. **(A, B)** Representative images of TOM20 by immunofluorescence staining in the indicated groups of mice raised for 12 wk after STZ injection. Scale bar = 50 μm.

We then measured the expression of the mitophagy-related proteins LC3, SQSTM1/p62 (sequestosome 1), PINK1, and BNIP3 by Western blot. As shown in Fig 3B–J, the expression of LC3-II and SQSTM1 in the renal cortex of STZ mice was increased, which suggested that diabetic mice had defective autophagy. VDR-KO aggravates this disorder, whereas pari treatment can alleviate the autophagy disorder. Meanwhile, the expression of BNIP3 and PINK1 decreased more dramatically in the KO-STZ group than in the WT-STZ group (Fig 3B and K). In addition, STZ led to serious declines in parkin RBR E3 ubiquitin protein ligase (Parkin/PRKN) and translocase of the outer membrane 20 (TOM20, also TOMM20) and the up-regulation of voltage-dependent anion channel 1 (VDAC1). VDR loss further exacerbated the decrease in Parkin and TOM20 and the increase in VDAC1 in STZ-induced mice. Paricalcitol effectively mitigated the loss of BNIP3 and PINK1/Parkin and the up-regulation of VDAC1 in STZ-induced mice compared with untreated mice (Figs 3B–J and S2A). Taken together, these data indicated that VDR deficiency worsens mitophagy defects mediated by BNIP3 and PINK1 in diabetic mice.

### VDR overexpression in TECs alleviates diabetic renal damage and fibrosis induced by STZ

**Figure 4.**
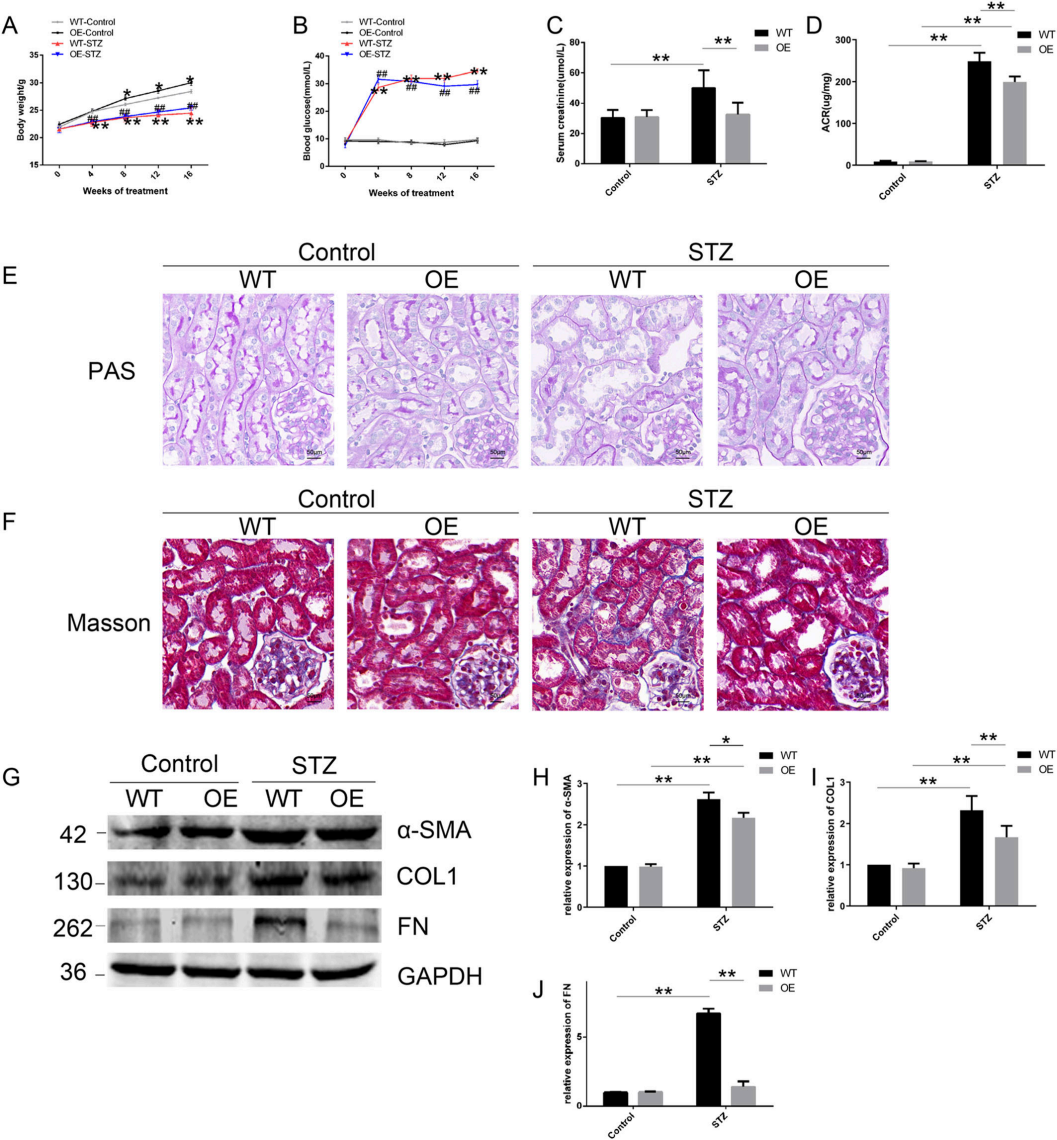
VDR overexpression in TECs reduces renal fibrosis in diabetic mice. **(A)** Body weight changes in WT, OE, and mice that received STZ treatment (WT-STZ, OE-STZ) within 16 wk. **(B)** Blood glucose levels in the indicated groups within 16 wk after STZ injections. Values are the mean ± SD. *P < 0.05, **P < 0.01 compared with the WT-control group and ^##^*P* < 0.01 compared with the OE-control group. **(C)** Serum creatinine values were assessed at 16 wk after STZ injections. **(D)** Urinary ACR values were tested at 16 wk after STZ injections. **(E, F)** Representative images of the PAS staining (E) and the Masson staining (F) at 16 wk after STZ injections. Scale bar = 50 μm. **(G, H, I, J)** Western blot analysis of α-SMA, COL1, and FN expression in the kidney of the indicated mice. Values are the mean ± SD. *P < 0.05, **P < 0.01. n = 5. Source data are available for this figure.

To further confirm the protective role of VDR in diabetic renal injury and fibrosis, transgenic mice with the TEC-specific overexpression of VDR were constructed, and this model was subjected to STZ injection to induce diabetes. VDR overexpression in TECs exerted no effects on body weight or blood glucose levels of diabetic mice (Fig 4A and B), whereas serum creatinine levels and proteinuria in diabetic mice were effectively reduced in the OE-STZ group compared with the WT-STZ group (Fig 4C and D). In addition, VDR overexpression in TECs not only alleviated renal tubular injury in diabetic mice but also effectively inhibited glomerular enlargement and tubulointerstitial fibrosis, as shown by the PAS and Masson staining of renal tissues (Fig 4E and F). The attenuated fibrosis in the VDR-OE STZ group was confirmed by the down-regulation of α-SMA, COL1, and FN compared with that in the WT-STZ group (Figs 4G–J and S1B). These data emphasized the renoprotective effect of VDR overexpression in TECs on diabetic mice.

### VDR overexpression in TECs attenuates mitophagy defects in STZ-induced diabetic mice

**Figure 5.**
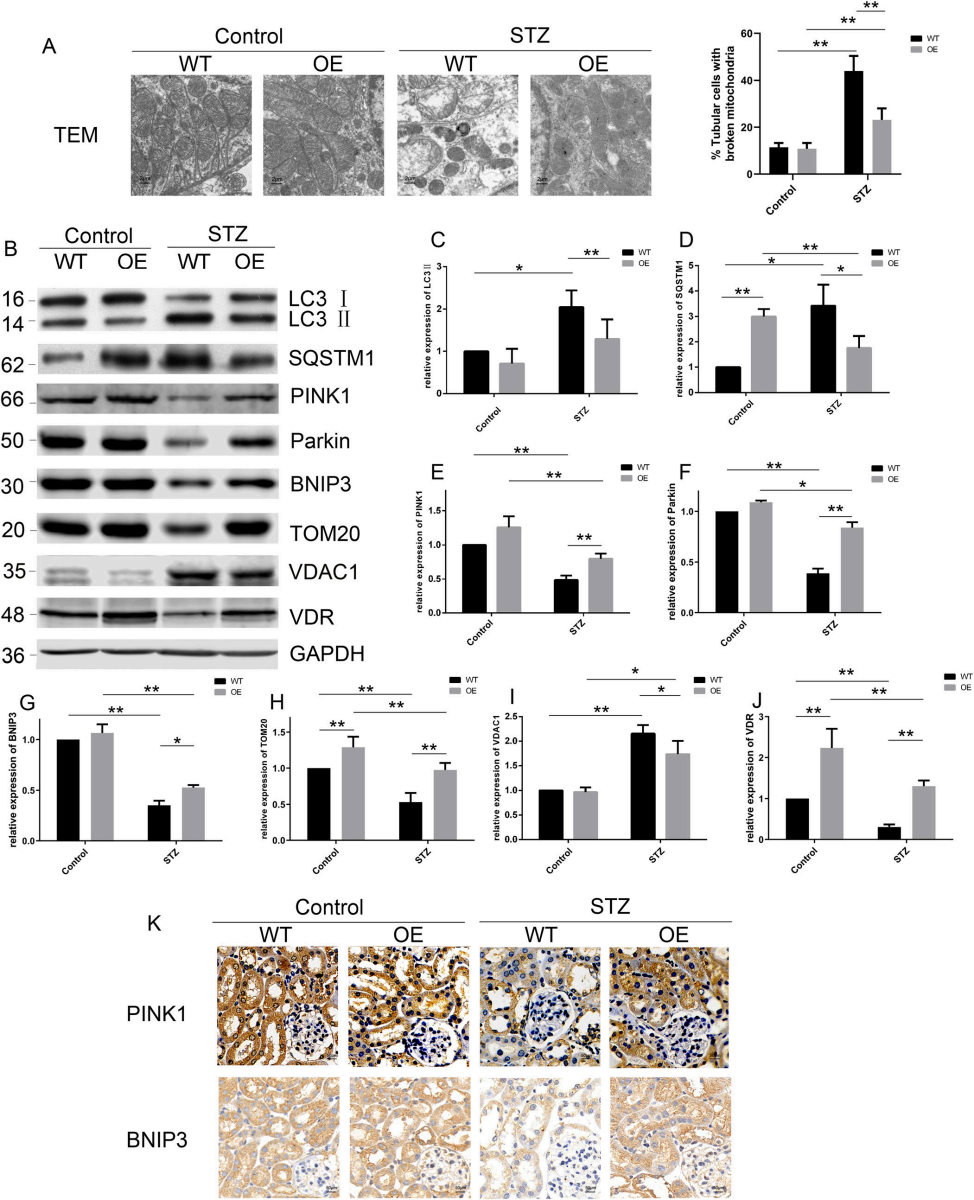
Overexpression of VDR in TECs attenuated the abnormality of mitophagy in diabetes. **(A)** Representative TEM images (left) of mitochondrial structures in TECs of WT-control, OE-control, WT-STZ, and OE-STZ mice at 12 wk after STZ injections and quantification of broken mitochondria (right). Scale bar = 2 μm. **(B, C, D, E, F, G, H, I, J)** Western blotting (B) and densitometric quantifications (C, D, E, F, G, H, I, J) of LC3, SQSTM1, PINK1, Parkin, BNIP3, TOM20, VDAC1, and VDR at 12 wk after STZ injections in the indicated groups. GAPDH was set as a loading control. The data are displayed as the mean ± SD, *P < 0.05, **P < 0.01. **(K)** Representative images of IHC staining of PINK1 and BNIP3 in the kidney at 12 wk after STZ injections. Scale bar = 50 μm. n = 5. Source data are available for this figure.

We subsequently examined mitophagy-related proteins in VDR-OE STZ-induced mice. As expected, the TEM images suggested that the STZ-induced chaotic mitochondrial morphology and enhanced swelling and fragmentation were significantly improved in VDR-OE mice (Fig 5A). In addition, IHC staining and Western blotting showed that the increased expression of LC3, SQSTM1, and VDAC1 and the decreased expression of PINK1, Parkin, BNIP3, and TOM20 were markedly reversed by the overexpression of VDR in TECs (Figs 5B–K and S2B). In summary, VDR agonist intervention and VDR overexpression effectively up-regulated the expression of PINK1 and BNIP3. These results suggest that VDR activation could alleviate mitochondrial morphological abnormalities and restore mitophagy by restoring the expression of PINK1 and BNIP3 in STZ-induced diabetic mice.

### VDR agonist (pari) ameliorated fibrosis and mitophagy in HK-2 cells under high glucose conditions

**Figure 6.**
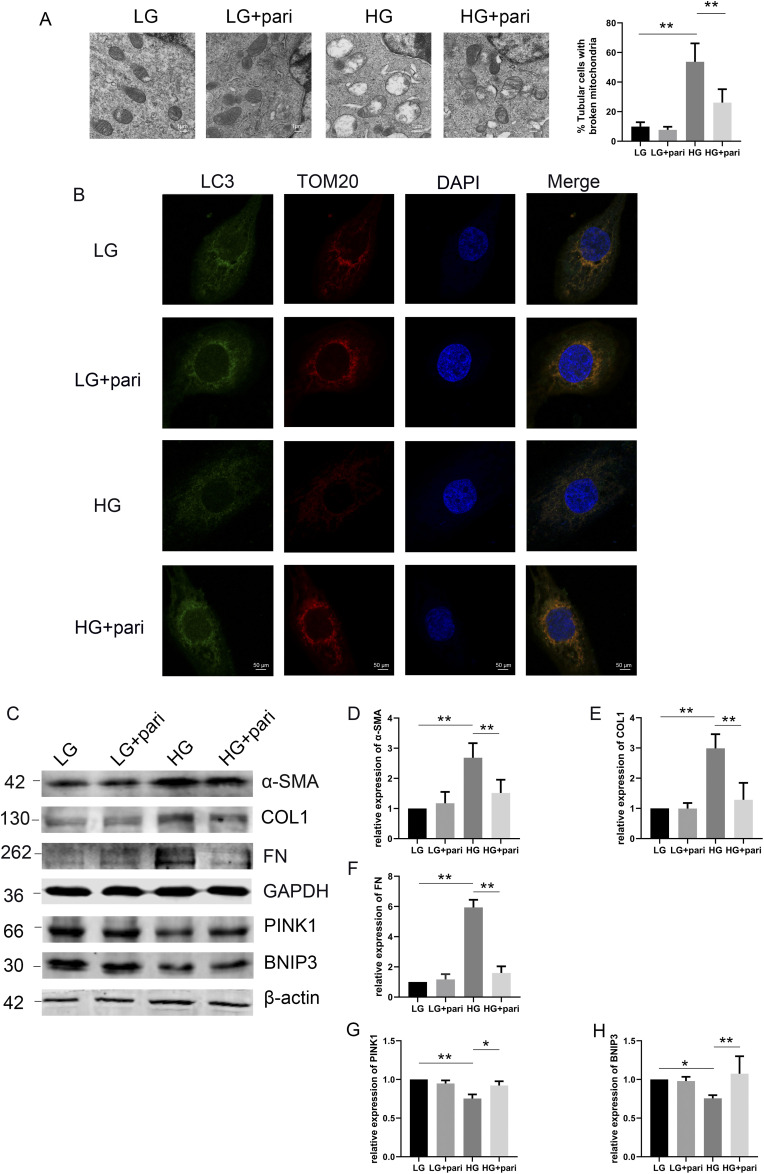
VD restored fibrosis and mitophagy induced by high glucose in HK-2 cells. **(A)** Representative TEM images of the indicated groups (left) and quantification of broken mitochondria (right). Scale bar = 1 μm. **(B)** HK-2 cells were collected for immunofluorescence of LC3 (autophagosomes, green) and TOM20 (mitochondria, red). Representative fluorescent images were captured by a confocal microscope. Scale bar = 50 μm. **(C)** Representative blots of α-SMA, COL1, FN, PINK1, and BNIP3 in HK-2 cells cultured with LG or HG medium. Meanwhile, the HG groups were added with pari (100 nM) for 48 h or not. GAPDH and β-actin were set as loading controls. **(D, E, F)** Densitometric quantifications of α-SMA, COL1, and FN. The data are presented as the mean ± SD from three independent experiments. **P < 0.01. **(G, H)** Densitometric quantifications of PINK1 and BNIP3 in HK-2 cells treated with pari or not. The data are expressed as the mean ± SD, *P < 0.05, **P < 0.01. All the experiments above were repeated at least three times. Source data are available for this figure.

Our above results indicated that renal fibrosis during diabetes was associated with mitophagy abnormalities. In vitro, we sought to determine whether paricalcitol treatment could effectively alleviate fibrosis and mitophagy defects induced by high glucose (HG) in HK-2 cells. HK-2 cells were cultured with high glucose (40 mM, 48 h), and paricalcitol (100 nM, 48 h) was added to treat HK-2 cells under HG conditions. As shown in the TEM images, mitochondrial cristae in HG-induced HK-2 cells were largely cracked or even missing, with outer mitochondrial membrane rupture or mitochondrial vacuolation. Paricalcitol effectively reduced mitochondrial swelling and the formation of mitochondrial vacuoles (Fig 6A).

Our immunofluorescence staining data showed that the overlap (yellow) fluorescence between LC3 (autophagosomes, green) and TOM20 (mitochondria, red) was greatly reduced (indicating defective mitophagy) in HK-2 cells with HG stimulation. Pari could promote the colocalization of mitochondria and autophagosomes in HK-2 cells (Fig 6B). Moreover, the expression of α-SMA, COL1, and FN was highly increased in the HG group compared with the low-dose glucose (LG, 5 mM) group. Paricalcitol treatment led to the down-regulation of α-SMA, COL1, and FN expression in HK-2 cells exposed to HG (Fig 6C–F). We then evaluated the expression of PINK1 and BNIP3 in HK-2 cells by Western blotting. Under HG conditions, the expression of PINK1 and BNIP3 in HK-2 cells was decreased, which could be improved by paricalcitol treatment (Fig 6C, G, and H). These results revealed that the VDR agonist could alleviate HG-induced fibrosis and mitophagy defects in HK-2 cells, which is consistent with the in vivo data.

### PINK1 and BNIP3 were involved in mitophagy and fibrosis in HG-treated HK-2 cells

**Figure 7.**
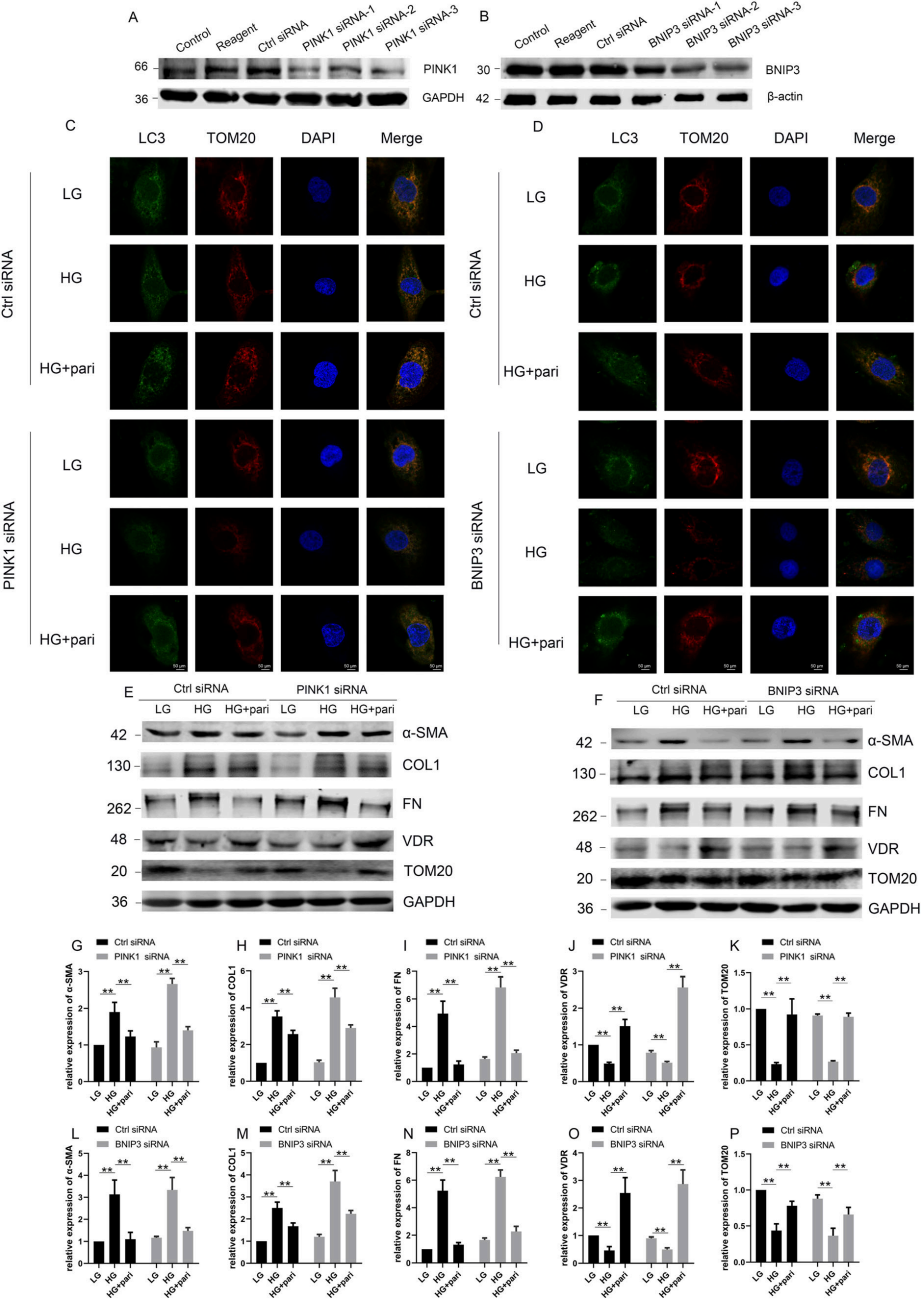
Effect of pari on mitophagy and fibrosis was largely mediated by PINK1 and BNIP3 in HG-treated HK-2 cells. **(A)** Western blot analysis for PINK1 expression in HK-2 with GAPDH as a loading control. HK-2 cells were transfected with transfection reagent, control siRNA, or three PINK1 siRNAs for 48 h. **(B)** Western blot analysis of BNIP3 expression in HK-2 with β-actin as a loading control. HK-2 cells were transfected with transfection reagent, control siRNA, or three BNIP3 siRNAs for 48 h. **(C)** HK-2 cells were divided into six groups: Ctrl siRNA+LG, Ctrl siRNA+HG, Ctrl siRNA+HG+pari (100 nM, 48 h), PINK1 siRNA+LG, PINK1 siRNA+HG, and PINK1 siRNA+HG+pari. HK-2 cells cultured in LG or HG medium were transfected with control or PINK1 siRNA for 48 h and subsequently collected for immunofluorescence of LC3 and TOM20. Representative images from three independent experiments were captured via confocal microscopy. Scale bar = 50 μm. **(D)** Cultured HK-2 cells were divided into six groups: Ctrl siRNA+LG, Ctrl siRNA+HG, Ctrl siRNA+HG+pari, BNIP3 siRNA+LG, BNIP3 siRNA+HG, and BNIP3 siRNA+HG+pari. Representative images from three independent experiments are shown. Scale bar = 50 μm. **(E, F)** Representative blots of α-SMA, COL1, FN, VDR, and TOM20 in HK-2 cells treated with the same as in C (E) or D (F). GAPDH was set as a loading control. **(E, F, G, H, I, J, K, L, M, N, O, P)** Densitometric quantifications of α-SMA, COL1, FN, VDR, and TOM20 in (E, F). The data are presented as the mean ± SD from three independent experiments. **P < 0.01. Ctrl = control. Source data are available for this figure.

Although the functions of PINK1 and BNIP3 have been widely recognized for their abilities to mediate mitophagy (25), whether they are directly involved in the regulation of VDR on mitophagy and fibrosis in diabetic conditions is unknown. Therefore, we transfected *PINK1* siRNAs or *BNIP3* siRNAs to knock down the expression of PINK1 or BNIP3 in HK-2 cells (Fig 7A and B). As shown by Western blot, the VDR level was decreased in HK-2 cells under HG conditions and pari specifically increased the protein level of VDR when PINK1 or BNIP3 was knocked down, respectively (Fig 7E, F, J, and O). The colocalization of LC3 and TOM20 expression was significantly increased in pari-treated control HK-2 cells with HG stimulation, and only slightly increased in pari-treated *PINK1* knockdown HK-2 cells (Fig 7C). BNIP3 knockdown likewise led to a similar decrease in the colocalization between LC3 and TOM20 in HG-treated HK-2 cells with pari treatment compared with the control siRNA group (Fig 7B and D). We also observed that pari treatment significantly attenuated the expression of α-SMA, COL1, and FN in HK-2 cells under HG stimulation by Western blotting, whereas the effect of pari on the expression of α-SMA, COL1, and FN was partly weakened when PINK1 was knocked down by siRNA transfection (Fig 7E and G–I). Meanwhile, inhibition of BNIP3 expression by BNIP3 siRNA resulted in a similar effect on fibrosis factors expression as PINK1 siRNA (Fig 7F and L–N).

**Figure 8.**
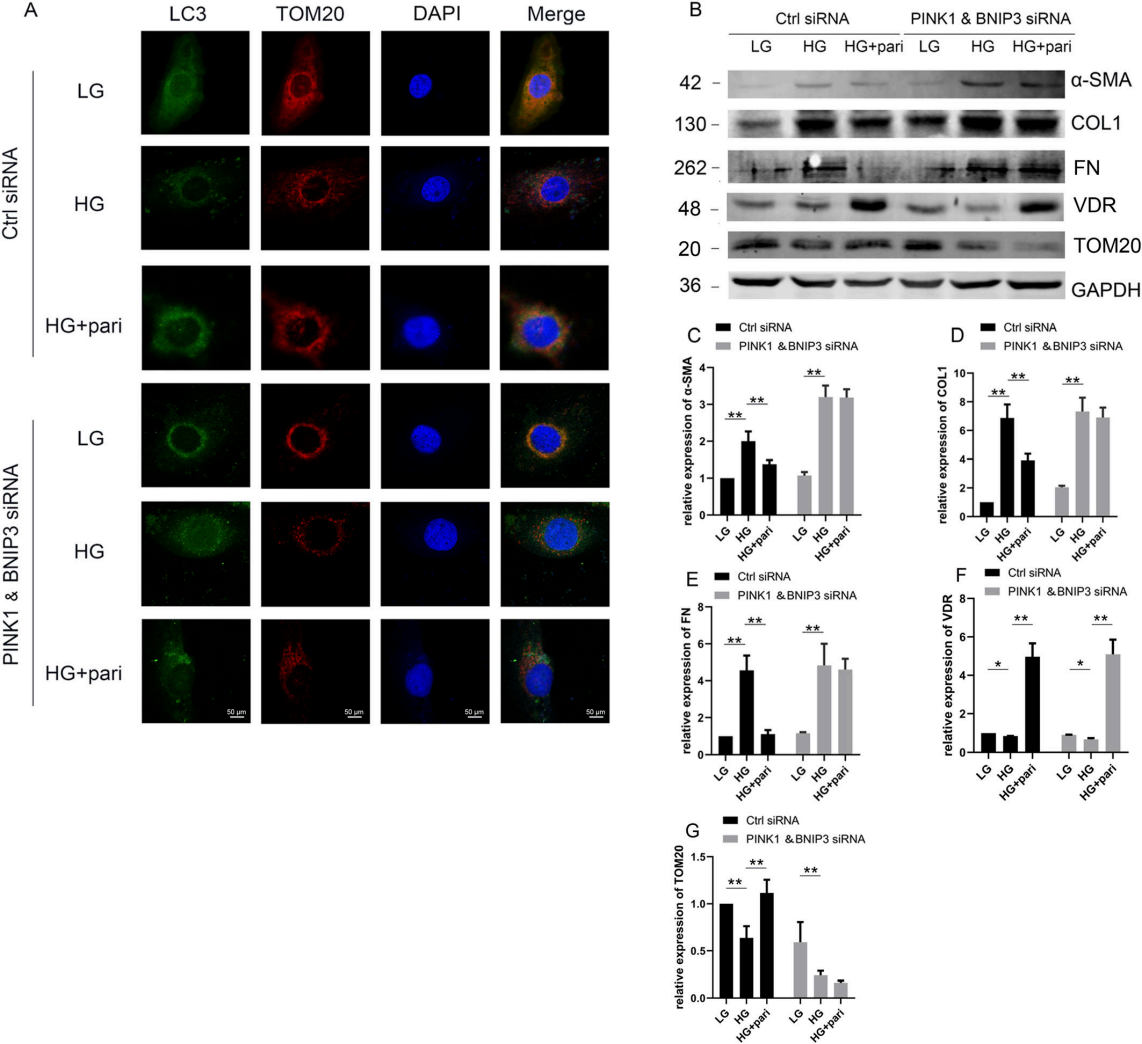
Pari could not improve mitophagy and fibrosis in HK-2 cells with PINK1 and BNIP3 siRNA transfection. Cultured HK-2 cells were divided into six groups: Ctrl siRNA+LG, Ctrl siRNA+HG, Ctrl siRNA+HG+pari, PINK1 and BNIP3 siRNA+LG, PINK1 and BNIP3 siRNA+HG, and PINK1 and BNIP3 siRNA+HG+pari. **(A)** Representative fluorescent images for LC3 and TOM20 captured from three independent experiments. Scale bar = 50 μm. **(B)** Representative blots of α-SMA, COL1, FN, VDR, and TOM20 in HK-2 cells. GAPDH was set as a loading control. **(B, C, D, E, F, G)** Densitometric quantifications of α-SMA, COL1, FN, VDR, and TOM20 in (B). The data are presented as the mean ± SD from three independent experiments. **P < 0.01. Ctrl = control. Source data are available for this figure.

Subsequently, we knocked down PINK1 and BNIP3 at the same time, and found that pari still significantly increased the expression of VDR in HK-2 cells even with HG stimulation (Fig 8B and F). However, pari could not increase the colocation of LC3 and TOM20 in HK-2 cells under HG conditions (Fig 8A), nor could it prevent HG-induced increase in fibrosis factors including α-SMA, COL1, and FN (Fig 8B–E). Pari can still increase the expression of TOM20 when inhibiting the expression of PINK1 or BNIP3 alone in HK-2 cells via siRNAs (Fig 7E, F, K, and P), but it cannot increase the level of TOM20 when inhibiting PINK1 and BNIP3 at the same time (Fig 8B and G), which indicates that pari may indirectly affect the expression of TOM20 by affecting the expression of PINK1 and BNIP3. These observations suggested that VDR regulates mitophagy via affecting the expression of PINK1 and BNIP3 in HG-treated HK-2 cells.

### VDR modulates PINK1 and BNIP3 via direct transcriptional regulation

**Figure 9.**
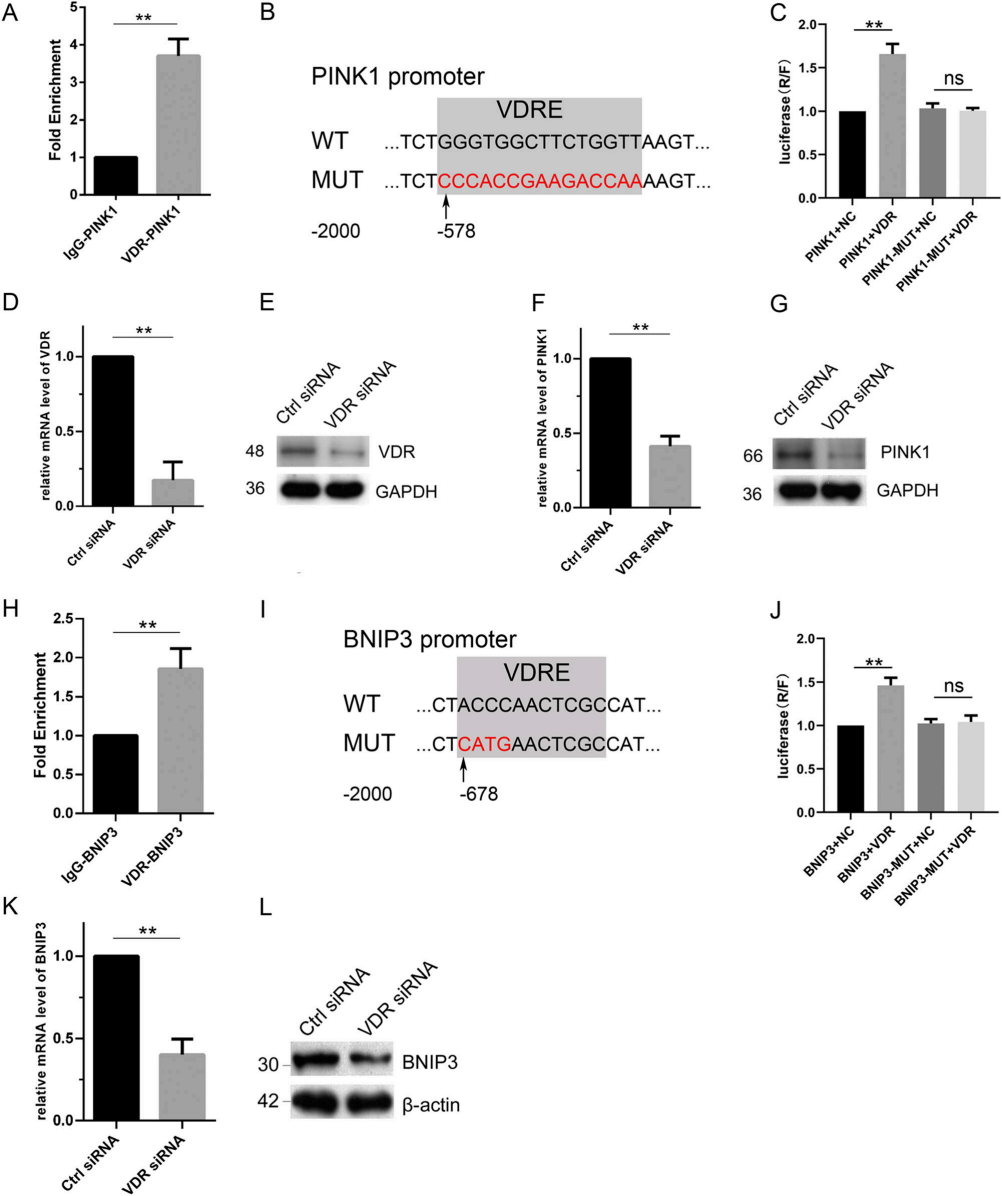
VDR could transcriptionally regulate the expression of PINK1 and BNIP3. **(A, H)** ChIP-qPCR analysis between VDR and *Pink1* gene or *Bnip3* gene in mouse renal tubular epithelial cells. **(B, I)** Sequences of the WT and mutated VDRE in the luciferase reporter gene vectors. **(C, J)** Luciferase reporter assay between VDR and *Pink1* gene or *Bnip3* gene in HEK-293T cells. **(D, E)** Relative mRNA level (D) and representative blot of VDR (E) in HK-2 with VDR siRNA or control siRNA plasmid transfection for 48 h. **(F, G)** Relative mRNA level (F) and representative blot of PINK1 (G) in HK-2 with VDR siRNA or control siRNA plasmid transfection for 48 h. GAPDH was used as a loading control. **(C, D, K, L)** Relative mRNA level (C) and representative blot (D) of BNIP3 with the down-regulation of VDR. β-Actin was set as a loading control. The data are displayed as the mean ± SD; *t* test was used between the two groups. **P < 0.01. Ctrl = control. NC = negative control. ns, no significance. All the experiments were repeated at least three times.

VDR is a classical nuclear transcription factor that targets numerous genes for transcriptional regulation (26). It is unclear whether VDR affects the expression of PINK1 and BNIP3 by transcriptional regulation. Here, we isolated DNA that can specifically bind to the VDR antibody in murine TECs by chromatin immunoprecipitation (ChIP) assays and then identified the sequences of PINK1 and BNIP3 that were immunoprecipitated by qPCR, thus confirming the interaction between VDR and the target genes *Pink1* and *Bnip3* (Fig 9A and H). Subsequently, to determine the specific regulatory sites, we constructed vectors including the promoter regions of PINK1 or BNIP3 (WT) and mutant vectors (MUT) that were mutated at the predicted VDR element and used luciferase reporter assays to monitor binding activity (Fig 9B and I). As shown in Fig 9C and J, VDR could increase luciferase activity in both WT *Pink1* and WT *Bnip3* groups, whereas VDR could not work in the mutated group. In addition, inhibition of VDR expression by VDR-specific siRNA in HK-2 cells decreased PINK1 and BNIP3 expression (Fig 9D–G, K, and L). This evidence suggests that the expression of PINK1 and BNIP3 was transcriptionally regulated by VDR.

## Discussion

In this study, we showed that VDR could ameliorate renal fibrosis in STZ-induced diabetic mice by up-regulating the expression of BNIP3 and PINK1. In VDR-KO and VDR-OE mice, we separately established the diabetic model induced by STZ and found that VDR deficiency was involved in mitophagy defects and fibrosis in STZ-induced mice. Furthermore, our data verified that VDR could directly regulate the expression of BNIP3 and PINK1, which are major mitophagy proteins.

DKD is related to many pathogenic factors, including hyperglycemia, hypoxia, and oxidative stress (27, 28), which can cause renal interstitial damage, such as mitochondrial dysfunction and tubulointerstitial fibrosis (5). Because these pathological changes are strongly associated with the progression of DKD, this study focused on the molecular mechanism of renal interstitial changes during the progression of diabetic renal injury. Mitochondrial dynamics, including mitophagy, play vital roles in DKD. An oxidative rush in damaged mitochondria and insufficient energy supplies lead to the progression of DKD (29). In response to advanced glycation end products, abnormal hemodynamics, and inflammation, mitochondrial function in the TECs of DN patients is more vulnerable to stress (9, 12, 30). Zhan M et al demonstrated that STZ-induced diabetic mice exhibited decreased mitochondrial production, oxidative dysfunction, increased mitochondrial fission, and damaged mitochondrial accumulation in kidney tissues (12). In our present study, we showed that there were a large number of damaged mitochondria that could not be effectively removed by autophagy in the TECs of STZ-induced diabetic mice, indicating mitophagy deficiency in DKD, which is consistent with Zuo’s report (31).

Mitophagy is a type of selective autophagy that removes damaged mitochondria to maintain the mitochondrial levels of cells and ensure mitochondrial quality control (32). Generally, mitophagy is mostly mediated by two different signaling pathways: the receptor-independent pathway (PINK1/Parkin signaling) and receptor-dependent pathways, including the BNIP3, Bcl-2/E1B-19K–interacting protein 3-like (BNIP3L/NIX), and FUN14 domain containing 1 (FUNDC1) pathways (13). Among those, the PINK1-mediated pathway has been reported most frequently. In DKD, the status of autophagy, including mitophagy, has been somewhat controversial. Xiao et al reported mitophagy inhibition with down-regulated LC3II and PINK1/Parkin expression in db/db mice and HG-induced HK-2 cells (33). Huang et al showed increased LC3II and SQSTM1 expression and up-regulated BNIP3 levels in STZ-injected rats and HG-treated HK-2 cells, indicating overactivated mitophagy in DKD (34). Our previous study showed elevated LC3II and SQSTM1 levels in STZ-induced C57 mice but defective autophagic flux in diabetic kidneys (23). Along with the down-regulation of BNIP3 and PINK1 expression, our results indicated that the autophagy-dependent clearance of damaged mitochondria was deficient in DKD mice induced by STZ, and this defective mitophagy mainly resulted from the decreases of BNIP3 and PINK1.

VDR is a classic nuclear transcription factor that is involved in inflammation, immune responses, tumorigenesis, autophagy, pyroptosis, and ferroptosis (17, 18, 22, 23, 35, 36). Vitamin D can specifically activate the expression of VDR. Moreover, vitamin D can bind with VDR, and the VD/VDR pathway mediates the series of downstream reactions mentioned above (20, 37). Here, using a VDR-KO mouse model and a transgenic mouse model with TEC-specific VDR overexpression, we further confirmed the role of VD/VDR signaling in diabetic renal fibrosis. VDR-KO exacerbated the impairment of mitophagy with promoted renal impairment and fibrosis in diabetic mice, whereas VDR activation with the agonist paricalcitol or TEC-specific VDR overexpression markedly restored the expression of BNIP3 and PINK1, attenuated mitochondrial morphology, and alleviated renal fibrosis. These data collectively indicate that VDR plays a renoprotective role by targeting mitophagy in DN. This protective effect was dependent on the regulation of PINK1 and BNIP3. Our gene sequencing and bioinformatics analysis results suggested that PINK1 and BNIP3 were transcriptional regulation targets of VDR, and our ChIP assays and luciferase reporter assays have confirmed this regulatory mechanism.

Our data showed that neither VDR overexpression nor a VDR agonist could up-regulate BNIP3 levels above the baseline level (WT as the control) but partly restored its expression under diabetic conditions. This restoration of BNIP3 was considered protective, which is consistent with Zheng’s report in STZ-induced diabetic rat model (38) and acute kidney injury mouse model induced by renal ischemia/reperfusion injury (39).

Renal tubulointerstitial fibrosis is an important injury manifestation in the progress of DKD, and anti-fibrosis therapy has always been an important strategy to delay the progress of DKD (40). VDR has long been reported to have an anti-fibrosis effect, whereas the mechanisms were not fully clarified (41). We have also previously found that VDR can affect inflammation by regulating autophagy in diabetic mice (23). Our present study prolonged the feeding time of diabetic mice, which also observed fibrosis damage that had not appeared in our previous studies. The results showed that VDR protected against renal fibrosis in STZ mice. Not only did the synthesis of extracellular matrix FN and COL1 decrease in the renal interstitium of STZ mice treated with VDR, but also the decrease in α-SMA production was detected, which means that the subsequent ECM deposition will decrease, which should also be one of the reasons for the decrease of fiber production in kidney tissues of STZ mice treated with VDR. This shows that VDR can not only play an anti-fibrosis role by inhibiting inflammation, but also has other mechanisms involved. For the first time, our study found that VDR can not only restore mitophagy, but also regulate two key mitophagy factors transcriptionally, PINK1 and BNIP3, which has not been reported before.

An RCT study showed that vitamin D and RAS inhibitors could further reduce albuminuria in DN patients (42). Our current study also suggested that VD/VDR could alleviate renal injury in diabetic mice (23). However, to date, there has been no report showing that vitamin D alone can effectively improve renal outcomes in DN patients. We hypothesize that this may be associated with the decreased expression of renal VDR and insufficient administration of VDR agonists. In vivo, WT diabetic mice were injected with paricalcitol (0.4 μg/kg, three times a week), and this vitamin D derivative effectively enhanced the expression of VDR and relieved kidney injury in diabetic mice. However, because of the multiple functions of vitamin D, such a large dose of vitamin D is bound to cause corresponding side effects in clinical patients that limit the use of VDR agonists. In the present study, we constructed TEC-specific VDR-overexpressing mice and observed significant renal protection mediated by VDR overexpression with or without a low dose of paricalcitol (0.1 μg/kg, three times per week). These results emphasized the protective role of VDR in DKD. Although gene therapy to specifically improve renal VDR expression in patients is not currently available, our research provides a new direction for DN treatment in the future.

In summary, the present study revealed defective mitophagy in STZ-induced diabetic kidneys and that VDR could ameliorate renal injury and fibrosis by restoring mitophagy through the PINK1 and BNIP3 pathways. This protective effect of VDR on mitophagy was related to the transcriptional regulation of PINK1 and BNIP3. In addition, we overexpressed VDR in TECs and combined low-dose VD injection to achieve promising DKD protection in a mouse model for the first time, providing new insights into VDR signaling in the prevention and treatment of DKD.

## Materials and Methods

### Mice

VDR-KO C57BL/6 mice and TEC-specific VDR overexpression (VDR-OE) transgenic C57BL/6 mice were generated by the Nanjing Institute of Biomedical Sciences. WT (*Vdr*^+/+^) and VDR-KO (*Vdr*^−/−^) mice were bred from heterozygous (*Vdr*^+/−^) mice. WT littermates were used as control for VDR specifically overexpression transgenic mice on TECs. Western blot, immunohistochemistry, and qPCR were used to determine the genotypes as previously described (17, 23). 8-wk-old male mice were injected with STZ (50 mg/kg, 5 d) to induce diabetes, and blood glucose values higher than 16.7 mmol/l (1 wk after STZ injection) were selected as eligible diabetic mice. Paricalcitol (0.4 μg/kg in the *Vdr*^+/+^ group and 0.1 μg/kg in the OE group, three times per week) was injected into diabetic mice from 2 wk after STZ injection until the mice were euthanized. All mice were kept in the specific pathogen-free barrier of the Department of Laboratory Animals at Central South University, and the Institutional Animal Care and Use Committee of Central South University have approved the animal experiments in this study (number: 2018sydw0167).

### Cell culture and treatments

HK-2 cells were obtained from the Institute of Kidney Disease, Central South University. HEK-293T cells and mouse renal TECs were provided by HonorGene. HK-2 cells were cultured in DMEM/F12 medium (1:1) containing 10% FBS in six-well plates, and low glucose (5 mM) or high glucose (40 mM) was added to the wells and cocultured with the cells for 48 h. To evaluate the effect of pari on PINK1 or BNIP3, HK-2 cells were transfected with PINK1 or BNIP3 siRNAs (50 nM) and pari (100 nM) was added to the medium at the same time. After 48 h, HK-2 cells were collected for Western blots or immunofluorescence. HEK-293T cells and murine TECs were maintained in DMEM supplemented with 10% FBS. VDR siRNA (HG-Si000376) was purchased from HonorGene. Control siRNA, PINK1, and BNIP3 siRNAs were synthetized by Sangon Biotech. The sequence information of siRNAs is provided in Table 1. According to the manufacturer's protocol, siRNA transfection in HK-2 cells was performed with Lipofectamine 3000 transfection reagent (L3000075; Thermo Fisher Scientific). The transfection efficiency was evaluated by qPCR and Western blotting.

**Table 1.** Sequence information of siRNAs.

Name	Sequence (5′-3′)
PINK1 siRNA-1	F: CGGCUGGAGGAGUAUCUGAUATT
	R: UAUCAGAUACUCCUCCAGCCGTT
PINK1 siRNA-2	F: GUUCCUCGUUAUGAAGAACUATT
	R: UAGUUCUUCAUAACGAGGAACTT
PINK1 siRNA-3	F: GCCGCAAAUGUGCUUCAUCUATT
	R: UAGAUGAAGCACAUUUGCGGCTT
BNIP3 siRNA-1	F: GAACUGCACUUCAGCAAUAAUTT
	R: AUUAUUGCUGAAGUGCAGUUCTT
BNIP3 siRNA-2	F: GCUUCUGAAACAGAUACCCAUTT
	R: AUGGGUAUCGUUUCAGAAGCTT
BNIP3 siRNA-3	F: GCUCUCUCAUUUGCUGGCCAUTT
	R: AUGGCCAGCAAAUGAGAGAGCTT
VDR siRNA	F: CCUGCUCAGAUCACUGUAUTT
	R: AUACAGUGAUCUGAGCAGGTT
Control siRNA	F: UUCUCCGAACGUGUCACGUTT
	R: ACGUGACACGUUCGGAGAATT

F: forward; R: reverse

### Reagents

A Dual-Luciferase Reporter Assay kit (E1910 and E1960) was obtained from Promega Corporation. STZ (S01130) was purchased from Sigma-Aldrich. The anti-TOM20 antibody (sc-17764) and the anti-VDAC1 antibody (sc-390996) were purchased from Santa Cruz Biotechnology. Paricalcitol was provided by Professor Yanchun Li of the University of Chicago. The anti-collagen Ⅰ antibody (ab6308), anti-fibronectin antibody (ab2413), anti-PINK1 antibody (ab23707), anti-Parkin antibody (ab77924), anti-BNIP3 antibody (ab109362), and anti-VDR antibody (ab134826) were purchased from Abcam (Cambridge, UK). The anti-α-SMA antibody (19245) was purchased from Cell Signaling Technology.

### qPCR

Total RNA was extracted by TRIzol reagent (15596026), which was obtained from Thermo Fisher Scientific. The mRNA reverse transcription kit (CW2569) was purchased from CoWin BioSciences. We searched target gene sequences in the NCBI database and designed the primers with Primer5 software. The primers were synthesized by Sangon Biotech. Sequences of the qPCR primers used in this study are outlined in Table 2.

**Table 2.** Sequences of the qPCR primers.

Name	Sequence (5′-3′)
H-Pink1	F: TGACCTTTGCCCCTAACACGAG
	R: GTAACTGAACGTGCTGACCCAT
H-Bnip3	F: CGCAGACACCACAAGATACCAA
	R: GCCGACTTGACCAATCCCAT
H-Vdr	F: ACCCACCTGCTGAGAGACCCAA
	R: ACCTCAACCAACCCCTTAGACCC
H-actin	F: ACCCTGAAGTACCCCATCGAG
	R: AGCACAGCCTGGATAGCAAC
M-Pink1	F: CATCGCCTATGAAATCTTTGGG
	R: AATTTCAGGTTCTTCAGGGCTA
M-Bnip3	F: AAAGGGTGCGTGCGGGTTATC
	R: GGTGGACAGCAAGGCGAGAATC
M-U6	F: CTCGCTTCGGCAGCACA
	R: AACGCTTCACGAATTTGCGT

H: human; M: mouse; F: forward; R: reverse

### Western blot analysis

Renal tissues and HK-2 cells from the indicated groups were lysed with RIPA lysis buffer, containing a 1% protease inhibitor cocktail (P8340; Sigma-Aldrich) and 10% PMSF (ST505; Beyotime). The separated proteins were transferred to polyvinylidene fluoride membranes (Millipore), which were incubated with the indicated primary antibodies overnight at 4°C. The membranes were incubated with secondary antibodies and scanned with an Odyssey CLx infrared imaging system (LI-COR) on the second day. The band intensities in the images were measured using ImageJ software.

### PAS staining

Fresh renal tissues were fixed with 4% PFA for 24 h and then embedded in paraffin. After being dewaxed, the paraffin slices were dyed in periodic acid solution for 15 min, Schiff's reagent for 30 min in the dark, and hematoxylin for 5 min. Subsequently, the slides were dehydrated in absolute ethanol and sealed with neutral balsam. The slides were observed using a Nikon microscope. Two experimenters independently selected five visual fields in each group.

### Masson staining

Paraffin-embedded kidney sections were dewaxed in xylene, then hydrated in ethanol with different concentrations, and washed with distilled water. Subsequently, the slices were dyed in Harris hematoxylin for 5 min, Ponceau–acid fuchsin for 5 min, phosphomolybdic acid solution for 5 min, and 1% glacial acetic acid for 1 min, and dehydrated with 95% alcohol many times. After being dehydrated with anhydrous ethanol, the slices are transparent with xylene and sealed with neutral glue. Under the microscope, the collagen fibers in renal tissues are blue and the nucleus is blue-black.

### IHC staining

Paraffin kidney sections (2–3 μm) were deparaffinized, hydrated, and antigen repaired. Subsequently, the tissues were incubated with primary antibodies targeting PINK1 and BNIP3 and the secondary antibody. After incubation with the indicated antibodies, freshly prepared DAB solution was added to the sections, which were observed under a microscope. The positive color is brown-yellow.

### Immunofluorescence and confocal microscopy

HK-2 cells precultured on coverslips in six-well plates were fixed with 4% PFA for 30 min and blocked with normal donkey serum for subsequent immunofluorescence staining. Anti-TOMM20 antibody (ab283317; Abcam) 1:500 and anti-LC3B antibody (ab192890; Abcam) 1:200 were used for immunofluorescence. After the sections that finished incubation with the first antibodies and the second antibodies were dried, DAPI staining solution was added to restain the nuclei, and the cells were incubated at room temperature for 10 min in the dark. Then, an anti-fluorescence quenching reagent is added to seal the samples. Under the confocal microscope (Nikon), the nucleus stained by DAPI is blue.

### TEM

Fresh kidney tissues or HK-2 cells were removed and quickly placed into an electron microscope fixative at 4°C for 2–4 h. After being embedded, tissues were cut into ultrathin sections at a thickness of 60–80 nm. After being stained with lead citrate and a saturated alcohol solution containing 2% uranyl acetate, the slices were dried at room temperature overnight. Then, the slices were observed under a transmission electron microscope (Hitachi), and cells in proximal tubules of mice or HK-2 cells were chosen for analysis. To determine the degree of mitochondrial damage in cells, we selected the view at low magnification (×5,000, scale bars = 2 μm) under the microscope for observation, and at least eight pictures were selected for statistics in each group. Microscopically, mitochondria with discontinuous mitochondrial membranes are regarded as damaged mitochondria. In the control group, about 10–15% of the mitochondria were damaged, and STZ greatly aggravated this damage ratio. In HK-2 cells, we used the same method for analysis, and the difference was the magnification (scale bars = 1 μm).

### Bioinformatics analysis

Whole-gene sequencing was used to detect variants between WT and KO mice with an Illumina high-throughput sequencing technique. Analysis and comparison of the screened results above were performed by FastQC. We searched the sequences of genes from the PubMed website. Potential gene (*Pink1*, *Bnip3*, and others including *Tom20*) promoter binding sites of VDR or VDR elements were first predicted with the JASPAR database.

### ChIP-qPCR assay

Total DNA was extracted from murine TECs. VDR antibodies (ab109234; Abcam) or IgG was used as probes. We designed primers for the predicted binding sites between VDR and PINK1 (forward: CCTGGCTCAACGTCTCATCT, reverse: CTACCCGCTCATCCCTGCAT) or BNIP3 (forward: ATTCAGTCTGGTGACATGGCTCAG, reverse: GGGTCAGGTCACTAGAAGCAG). Then, quantitative PCR was performed with these primers. An enrichment factor greater than 1 indicated that the enrichment capacity of nonspecific antibody adsorption was less than that of specific antibody effects, and it was judged as a positive result.

### Dual-luciferase reporter assay

HEK-293T cells were transfected with the designed vectors in 24-well plates for 48 h using Lipofectamine 3000 transfection reagent. The pHG-PromDetect-musPINK1p vector (PINK1), pGL3-Basic-BNIP3 vector (BNIP3), pGL3-Basic-BNIP3 mutant vector (BNIP3 MUT), and pcDNA3.1(+)-VDR vector (VDR) were synthetized by Sangon Biotech. The pHG-PromDetect-musPINK1p mutant vector (PINK1 MUT) was synthetized by HonorGene. The activities of firefly and Renilla luciferase were measured by a GloMax 20/20 chemiluminescence detector (Promega).

### Statistical analysis

The data are presented as the means ± SD. Unpaired or paired *t* tests, one-way ANOVA, and two-way ANOVA were used for statistical comparisons between different groups and were performed by GraphPad Prism 8.0 software. A value of *P* < 0.05 was considered to be statistically significant.

## Supplementary Material

Reviewer comments

## References

[1] Tönnies T, Rathmann W, Hoyer A, Brinks R, Kuss O (2021) Quantifying the underestimation of projected global diabetes prevalence by the international diabetes federation (IDF) diabetes atlas. BMJ Open Diabetes Res Care 9: e002122. 10.1136/bmjdrc-2021-002122 PMC8370495 34400463

[2] Wu W, Huang XR, You Y, Xue L, Wang XJ, Meng X, Lin X, Shen J, Yu X, Lan HY, (2021) Latent TGF-β1 protects against diabetic kidney disease via Arkadia/Smad7 signaling. Int J Biol Sci 17: 3583–3594. 10.7150/ijbs.61647 34512167 PMC8416717

[3] Xue M, Cheng Y, Han F, Chang Y, Yang Y, Li X, Chen L, Lu Y, Sun B, Chen L (2018) Triptolide attenuates renal tubular epithelial-mesenchymal transition via the MiR-188-5p-mediated PI3K/AKT pathway in diabetic kidney disease. Int J Biol Sci 14: 1545–1557. 10.7150/ijbs.24032 30263007 PMC6158722

[4] Puglisi S, Rossini A, Poli R, Dughera F, Pia A, Terzolo M, Reimondo G (2021) Effects of SGLT2 inhibitors and GLP-1 receptor agonists on renin-angiotensin-aldosterone system. Front Endocrinol (Lausanne) 12: 738848. 10.3389/fendo.2021.738848 34745006 PMC8567993

[5] Liu XQ, Jiang L, Lei L, Nie ZY, Zhu W, Wang S, Zeng HX, Zhang SQ, Zhang Q, Yard B, (2020) Carnosine alleviates diabetic nephropathy by targeting GNMT, a key enzyme mediating renal inflammation and fibrosis. Clin Sci (Lond) 134: 3175–3193. 10.1042/CS20201207 33241846 PMC7726623

[6] Zeng LF, Xiao Y, Sun L (2019) A glimpse of the mechanisms related to renal fibrosis in diabetic nephropathy. Adv Exp Med Biol 1165: 49–79. 10.1007/978-981-13-8871-2_4 31399961

[7] Hasegawa K, Wakino S, Simic P, Sakamaki Y, Minakuchi H, Fujimura K, Hosoya K, Komatsu M, Kaneko Y, Kanda T, (2013) Renal tubular Sirt1 attenuates diabetic albuminuria by epigenetically suppressing Claudin-1 overexpression in podocytes. Nat Med 19: 1496–1504. 10.1038/nm.3363 24141423 PMC4041199

[8] Bhargava P, Schnellmann RG (2017) Mitochondrial energetics in the kidney. Nat Rev Nephrol 13: 629–646. 10.1038/nrneph.2017.107 28804120 PMC5965678

[9] Schiffer TA, Friederich-Persson M (2017) Mitochondrial reactive oxygen species and kidney hypoxia in the development of diabetic nephropathy. Front Physiol 8: 211. 10.3389/fphys.2017.00211 28443030 PMC5386984

[10] Pradeepkiran JA, Reddy PH (2020) Defective mitophagy in Alzheimer’s disease. Ageing Res Rev 64: 101191. 10.1016/j.arr.2020.101191 33022416 PMC7710581

[11] Tong M, Saito T, Zhai P, Oka SI, Mizushima W, Nakamura M, Ikeda S, Shirakabe A, Sadoshima J (2019) Mitophagy is essential for maintaining cardiac function during high fat diet-induced diabetic cardiomyopathy. Circ Res 124: 1360–1371. 10.1161/CIRCRESAHA.118.314607 30786833 PMC6483841

[12] Zhan M, Usman IM, Sun L, Kanwar YS (2015) Disruption of renal tubular mitochondrial quality control by Myo-inositol oxygenase in diabetic kidney disease. J Am Soc Nephrol 26: 1304–1321. 10.1681/ASN.2014050457 25270067 PMC4446875

[13] Shu L, Hu C, Xu M, Yu J, He H, Lin J, Sha H, Lu B, Engelender S, Guan M, (2021) ATAD3B is a mitophagy receptor mediating clearance of oxidative stress-induced damaged mitochondrial DNA. EMBO J 40: e106283. 10.15252/embj.2020106283 33665835 PMC8047441

[14] Song C, Zhang A, Zhang M, Song Y, Huangfu H, Jin S, Sun Y, Zhang C, Shi D, Wang J, (2023) Nrf2/PINK1-mediated mitophagy induction alleviates sodium fluoride-induced hepatic injury by improving mitochondrial function, oxidative stress, and inflammation. Ecotoxicol Environ Saf 252: 114646. 10.1016/j.ecoenv.2023.114646 36791501

[15] Zhang X, Feng J, Li X, Wu D, Wang Q, Li S, Shi C (2021) Mitophagy in diabetic kidney disease. Front Cell Dev Biol 9: 778011. 10.3389/fcell.2021.778011 34957109 PMC8703169

[16] Klionsky DJ, Petroni G, Amaravadi RK, Baehrecke EH, Ballabio A, Boya P, Bravo-San Pedro JM, Cadwell K, Cecconi F, Choi AMK, (2021) Autophagy in major human diseases. Embo j 40: e108863. 10.15252/embj.2021108863 34459017 PMC8488577

[17] Jiang S, Zhang H, Li X, Yi B, Huang L, Hu Z, Li A, Du J, Li Y, Zhang W (2021) Vitamin D/VDR attenuate cisplatin-induced AKI by down-regulating NLRP3/Caspase-1/GSDMD pyroptosis pathway. J Steroid Biochem Mol Biol 206: 105789. 10.1016/j.jsbmb.2020.105789 33259938

[18] Hu Z, Zhang H, Yi B, Yang S, Liu J, Hu J, Wang J, Cao K, Zhang W (2020) VDR activation attenuate cisplatin induced AKI by inhibiting ferroptosis. Cell Death Dis 11: 73. 10.1038/s41419-020-2256-z 31996668 PMC6989512

[19] Kang ZS, Wang C, Han XL, Du JJ, Li YY, Zhang C (2018) Design, synthesis and biological evaluation of non-secosteriodal vitamin D receptor ligand bearing double side chain for the treatment of chronic pancreatitis. Eur J Med Chem 146: 541–553. 10.1016/j.ejmech.2018.01.073 29407979

[20] Battistini C, Ballan R, Herkenhoff ME, Saad SMI, Sun J (2020) Vitamin D modulates intestinal microbiota in inflammatory bowel diseases. Int J Mol Sci 22: 362. 10.3390/ijms22010362 33396382 PMC7795229

[21] Huang HY, Lin TW, Hong ZX, Lim LM (2023) Vitamin D and diabetic kidney disease. Int J Mol Sci 24: 3751. 10.3390/ijms24043751 36835159 PMC9960850

[22] Yi B, Huang J, Zhang W, Li AM, Yang SK, Sun J, Wang JW, Li YC, Zhang H (2016) Vitamin D receptor down-regulation is associated with severity of albuminuria in type 2 diabetes patients. J Clin Endocrinol Metab 101: 4395–4404. 10.1210/jc.2016-1516 27552538

[23] Li A, Yi B, Han H, Yang S, Hu Z, Zheng L, Wang J, Liao Q, Zhang H (2022) Vitamin D-VDR (vitamin D receptor) regulates defective autophagy in renal tubular epithelial cell in streptozotocin-induced diabetic mice via the AMPK pathway. Autophagy 18: 877–890. 10.1080/15548627.2021.1962681 34432556 PMC9037529

[24] Springer MZ, Poole LP, Drake LE, Bock-Hughes A, Boland ML, Smith AG, Hart J, Chourasia AH, Liu I, Bozek G, (2021) BNIP3-dependent mitophagy promotes cytosolic localization of LC3B and metabolic homeostasis in the liver. Autophagy 17: 3530–3546. 10.1080/15548627.2021.1877469 33459136 PMC8632322

[25] Nguyen TT, Wei S, Nguyen TH, Jo Y, Zhang Y, Park W, Gariani K, Oh CM, Kim HH, Ha KT, (2023) Mitochondria-associated programmed cell death as a therapeutic target for age-related disease. Exp Mol Med 55: 1595–1619. 10.1038/s12276-023-01046-5 37612409 PMC10474116

[26] Carlberg C, Molnár F (2015) Vitamin D receptor signaling and its therapeutic implications: Genome-wide and structural view. Can J Physiol Pharmacol 93: 311–318. 10.1139/cjpp-2014-0383 25741777

[27] Friederich-Persson M, Thörn E, Hansell P, Nangaku M, Levin M, Palm F (2013) Kidney hypoxia, attributable to increased oxygen consumption, induces nephropathy independently of hyperglycemia and oxidative stress. Hypertension 62: 914–919. 10.1161/HYPERTENSIONAHA.113.01425 24019401 PMC3867444

[28] Nordquist L, Friederich-Persson M, Fasching A, Liss P, Shoji K, Nangaku M, Hansell P, Palm F (2015) Activation of hypoxia-inducible factors prevents diabetic nephropathy. J Am Soc Nephrol 26: 328–338. 10.1681/ASN.2013090990 25183809 PMC4310648

[29] Saxena S, Mathur A, Kakkar P (2019) Critical role of mitochondrial dysfunction and impaired mitophagy in diabetic nephropathy. J Cell Physiol 234: 19223–19236. 10.1002/jcp.28712 31032918

[30] Nair V, Komorowsky CV, Weil EJ, Yee B, Hodgin J, Harder JL, Godfrey B, Ju W, Boustany-Kari CM, Schwarz M, (2018) A molecular morphometric approach to diabetic kidney disease can link structure to function and outcome. Kidney Int 93: 439–449. 10.1016/j.kint.2017.08.013 29054530 PMC5794609

[31] Zuo Z, Jing K, Wu H, Wang S, Ye L, Li Z, Yang C, Pan Q, Liu WJ, Liu HF (2020) Mechanisms and functions of mitophagy and potential roles in renal disease. Front Physiol 11: 935. 10.3389/fphys.2020.00935 32903665 PMC7438724

[32] Gustafsson ÅB, Dorn GW 2nd (2019) Evolving and expanding the roles of mitophagy as a homeostatic and pathogenic process. Physiol Rev 99: 853–892. 10.1152/physrev.00005.2018 30540226 PMC6442924

[33] Xiao L, Xu X, Zhang F, Wang M, Xu Y, Tang D, Wang J, Qin Y, Liu Y, Tang C, (2017) The mitochondria-targeted antioxidant MitoQ ameliorated tubular injury mediated by mitophagy in diabetic kidney disease via Nrf2/PINK1. Redox Biol 11: 297–311. 10.1016/j.redox.2016.12.022 28033563 PMC5196243

[34] Huang C, Zhang Y, Kelly DJ, Tan CY, Gill A, Cheng D, Braet F, Park JS, Sue CM, Pollock CA, (2016) Thioredoxin interacting protein (TXNIP) regulates tubular autophagy and mitophagy in diabetic nephropathy through the mTOR signaling pathway. Sci Rep 6: 29196. 10.1038/srep29196 27381856 PMC4933928

[35] Li A, Zhang W, Zhang H, Yi B (2017) [Vitamin D/vitamin D receptor, autophagy and inflammation relevant diseases]. Zhong Nan Da Xue Xue Bao Yi Xue Ban 42: 979–985. 10.11817/j.issn.1672-7347.2017.08.017 28872092

[36] Voutsadakis IA (2020) Vitamin D receptor (VDR) and metabolizing enzymes CYP27B1 and CYP24A1 in breast cancer. Mol Biol Rep 47: 9821–9830. 10.1007/s11033-020-05780-1 33259013

[37] Carlberg C, Muñoz A (2022) An update on vitamin D signaling and cancer. Semin Cancer Biol 79: 217–230. 10.1016/j.semcancer.2020.05.018 32485310

[38] Jin L, Zheng D, Yang G, Li W, Yang H, Jiang Q, Chen Y, Zhang Y, Xie X (2020) Tilapia skin peptides ameliorate diabetic nephropathy in STZ-induced diabetic rats and HG-induced GMCs by improving mitochondrial dysfunction. Mar Drugs 18: 363. 10.3390/md18070363 32679664 PMC7401261

[39] Fu ZJ, Wang ZY, Xu L, Chen XH, Li XX, Liao WT, Ma HK, Jiang MD, Xu TT, Xu J, (2020) HIF-1α-BNIP3-mediated mitophagy in tubular cells protects against renal ischemia/reperfusion injury. Redox Biol 36: 101671. 10.1016/j.redox.2020.101671 32829253 PMC7452120

[40] Tang G, Li S, Zhang C, Chen H, Wang N, Feng Y (2021) Clinical efficacies, underlying mechanisms and molecular targets of Chinese medicines for diabetic nephropathy treatment and management. Acta Pharm Sin B 11: 2749–2767. 10.1016/j.apsb.2020.12.020 34589395 PMC8463270

[41] Song Z, Xiao C, Jia X, Luo C, Shi L, Xia R, Zhu J, Zhang S (2021) Vitamin D/VDR protects against diabetic kidney disease by restoring podocytes autophagy. Diabetes Metab Syndr Obes 14: 1681–1693. 10.2147/DMSO.S303018 33889003 PMC8057803

[42] de Zeeuw D, Agarwal R, Amdahl M, Audhya P, Coyne D, Garimella T, Parving HH, Pritchett Y, Remuzzi G, Ritz E, (2010) Selective vitamin D receptor activation with paricalcitol for reduction of albuminuria in patients with type 2 diabetes (VITAL study): A randomised controlled trial. Lancet 376: 1543–1551. 10.1016/S0140-6736(10)61032-X 21055801

