# Supplementary figures and images for: VDR restores the expression of PINK1 and BNIP3 in TECs of streptozotocin-induced diabetic mice
Source: Life Sci Alliance. 2024 May 2;7(7):e202302474. doi: 10.26508/lsa.202302474 (PMC11066303; doi:10.26508/lsa.202302474)

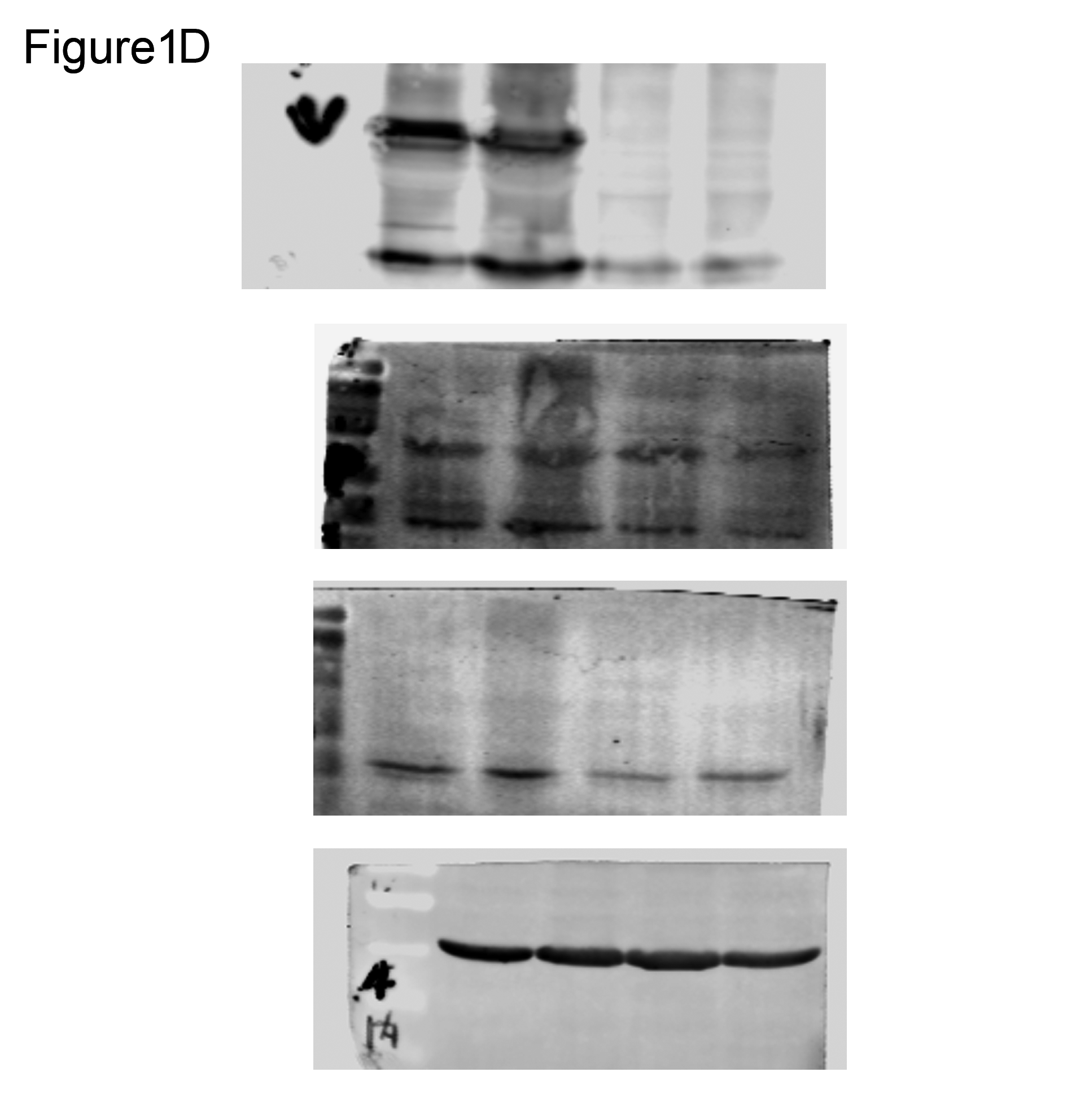

Supplement: Supplementary file 1 [file LSA-2023-02474_SdataF1.tif]

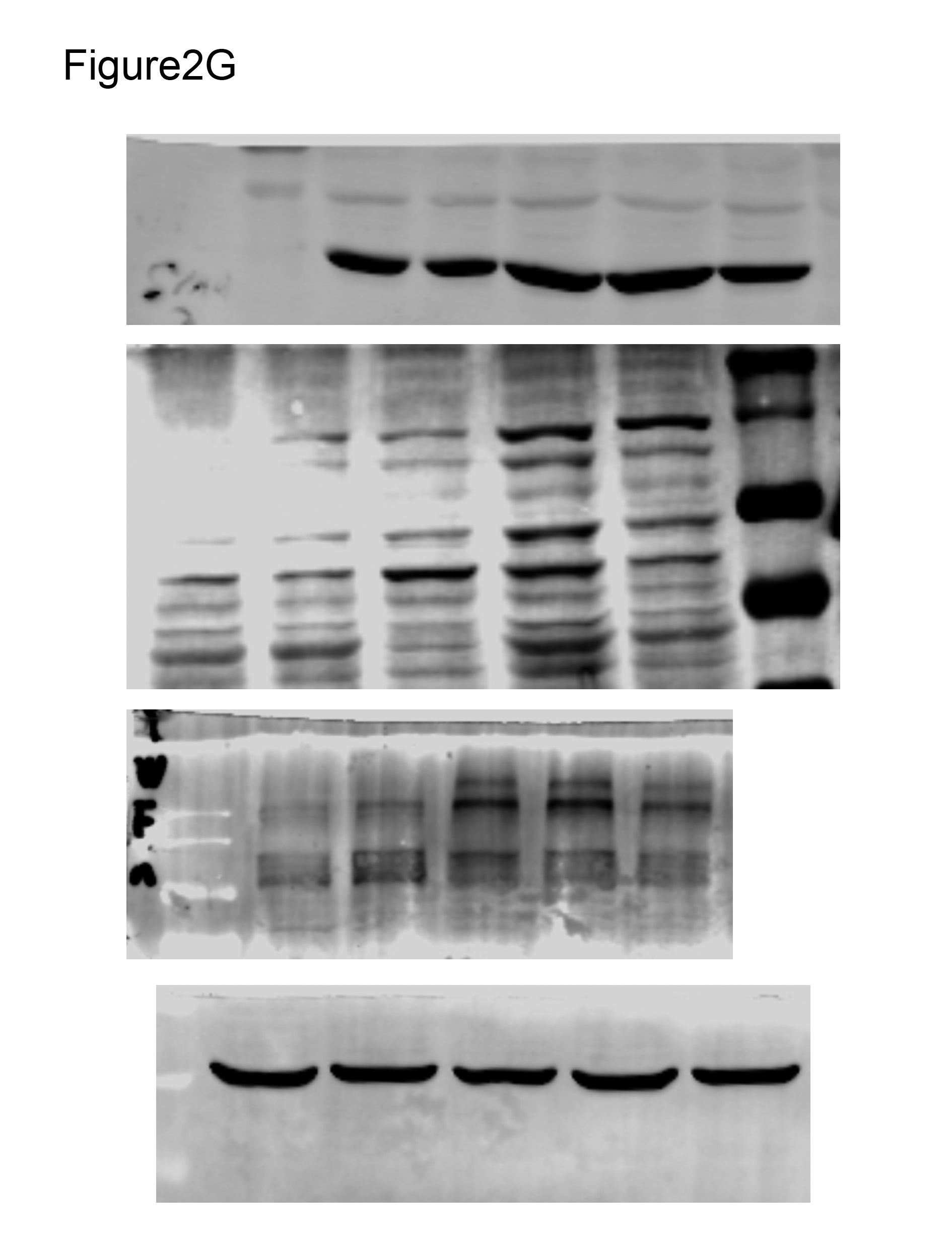

Supplement: Supplementary file 2 [file LSA-2023-02474_SdataF2.tif]

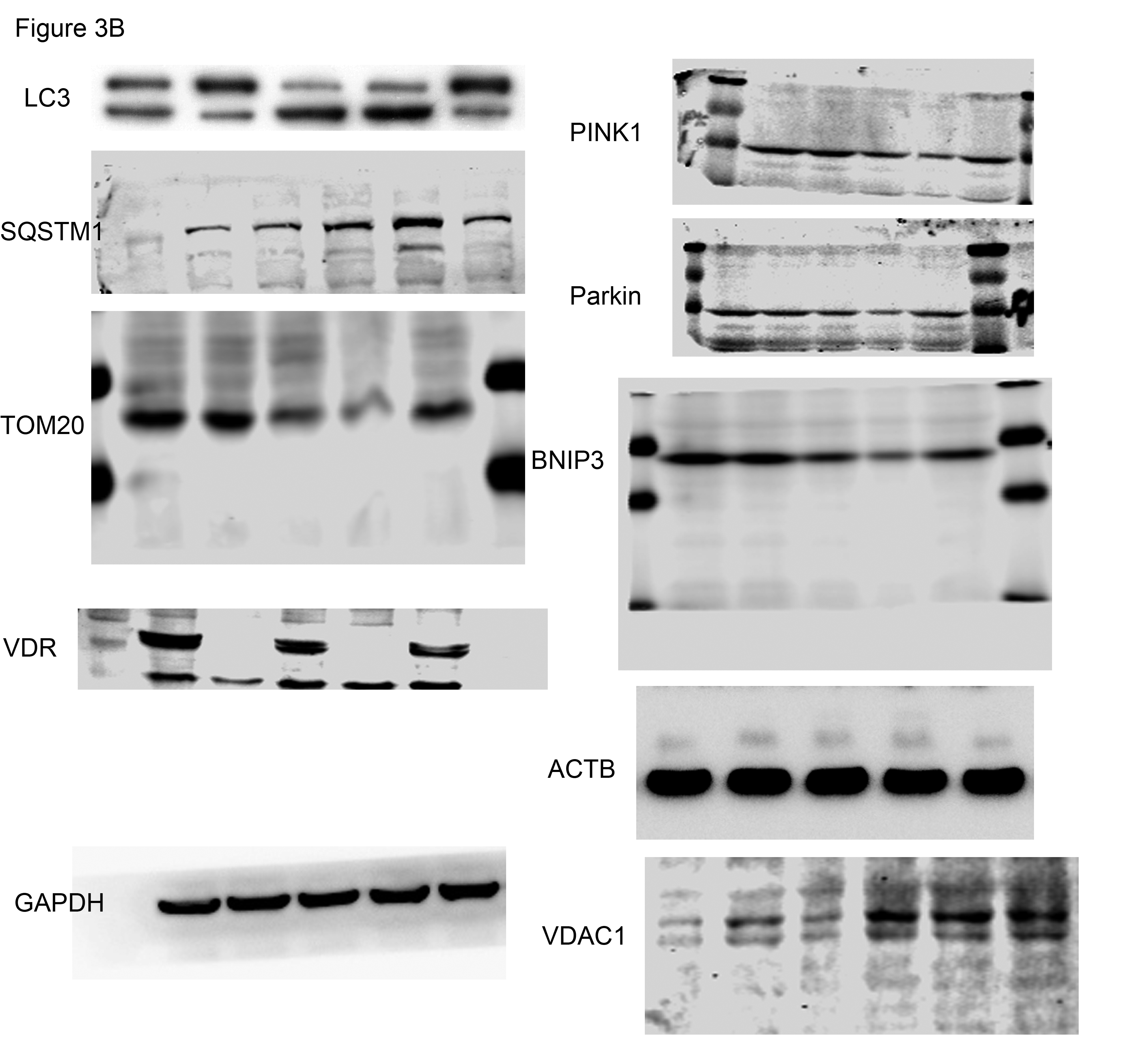

Supplement: Supplementary file 3 [file LSA-2023-02474_SdataF3.1.tif]

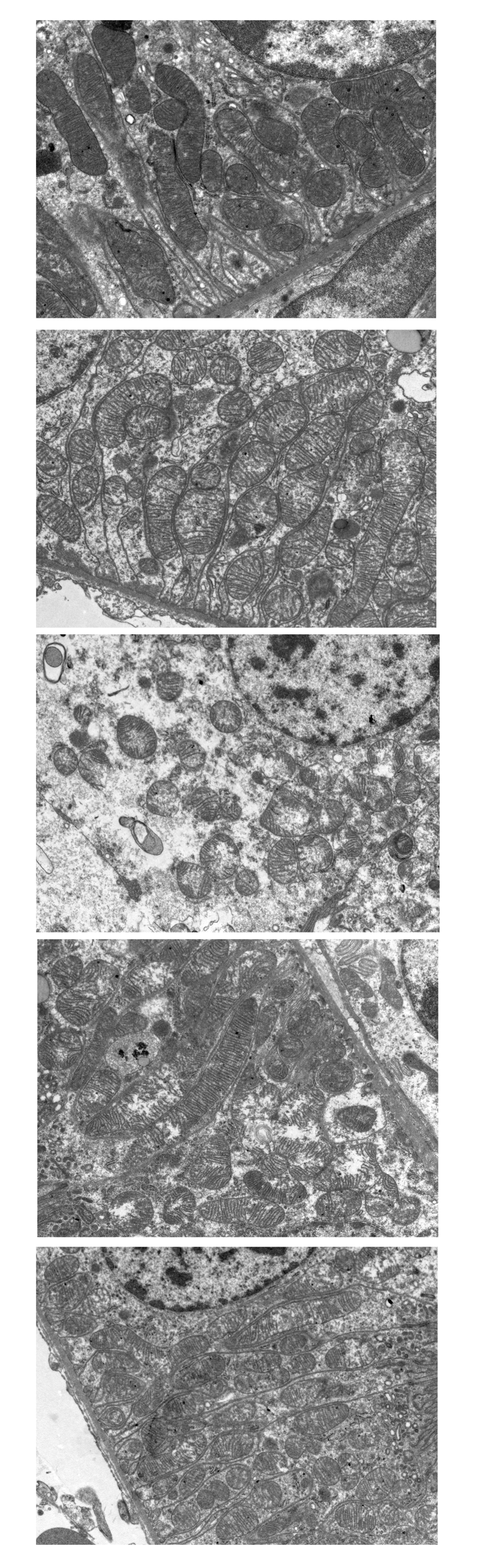

Supplement: Supplementary file 4 [file LSA-2023-02474_SdataF3.2.tif]

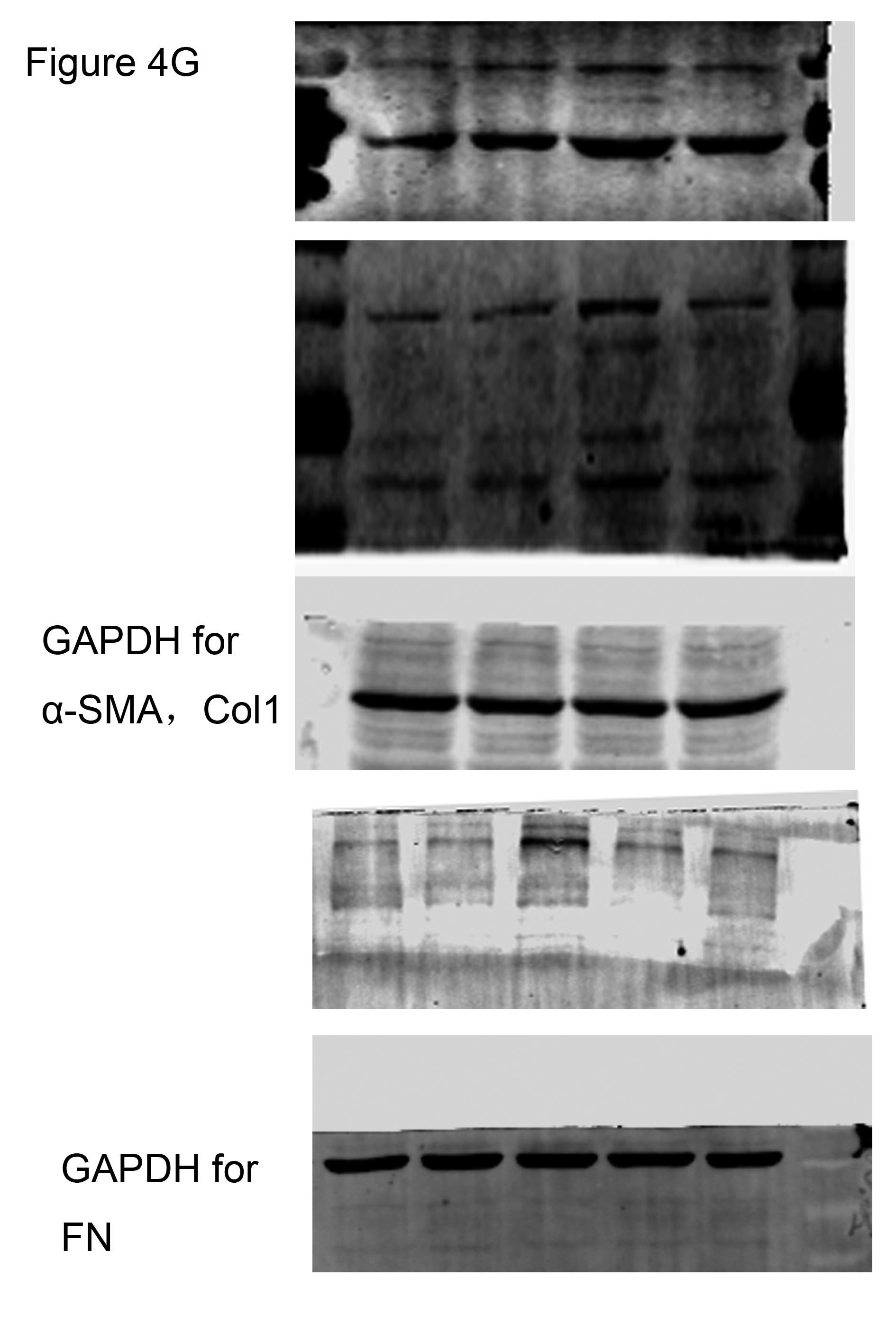

Supplement: Supplementary file 5 [file LSA-2023-02474_SdataF4.tif]

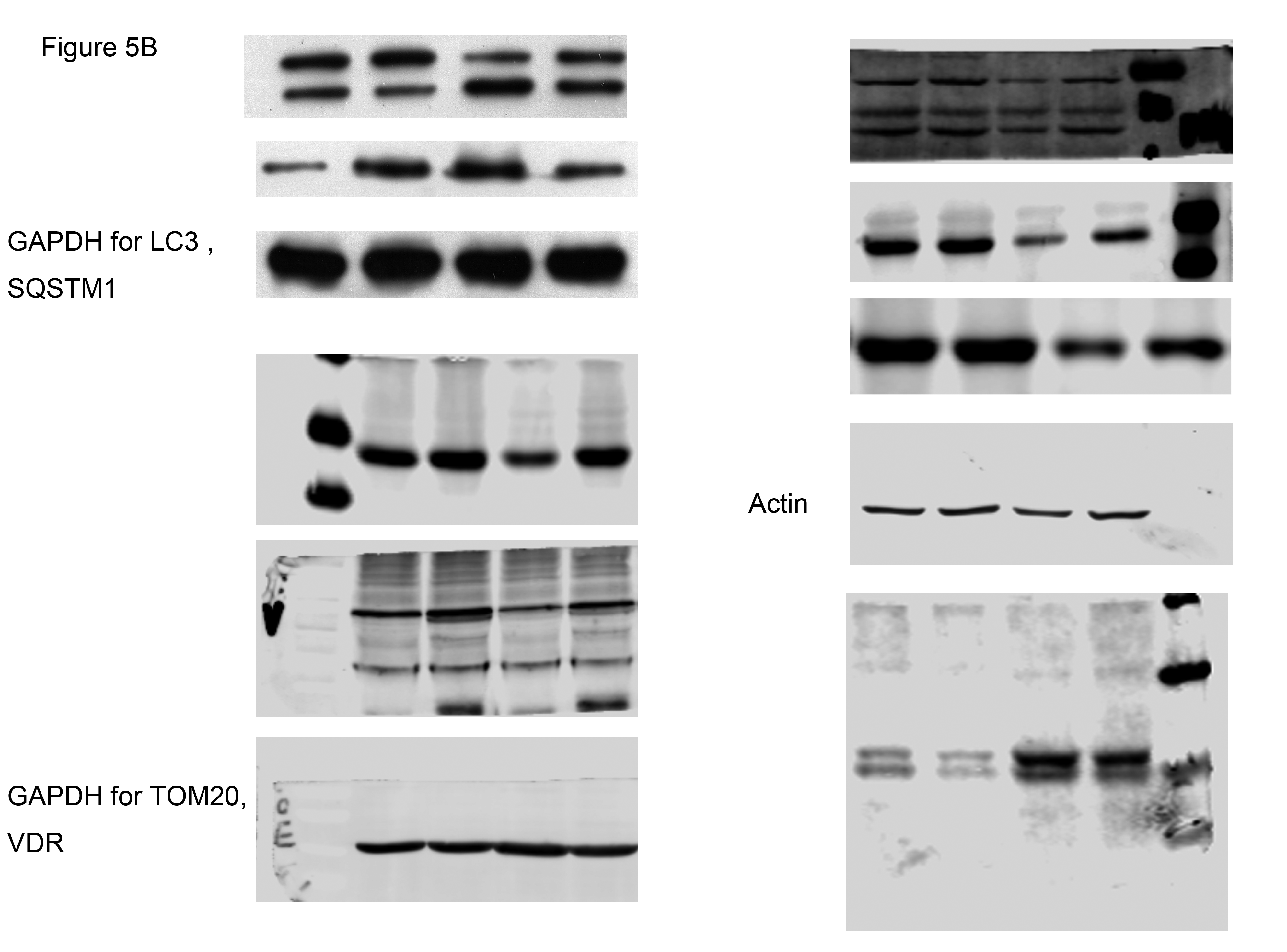

Supplement: Supplementary file 6 [file LSA-2023-02474_SdataF5.1.tif]

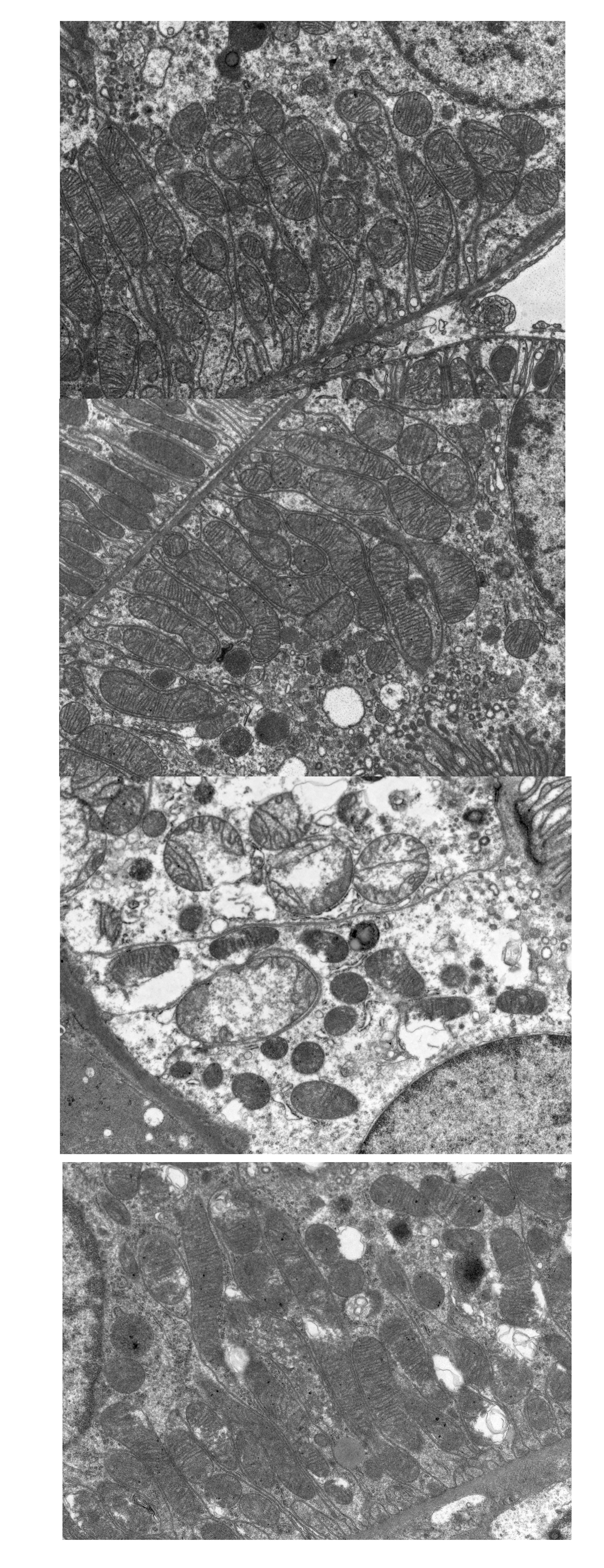

Supplement: Supplementary file 7 [file LSA-2023-02474_SdataF5.2.tif]

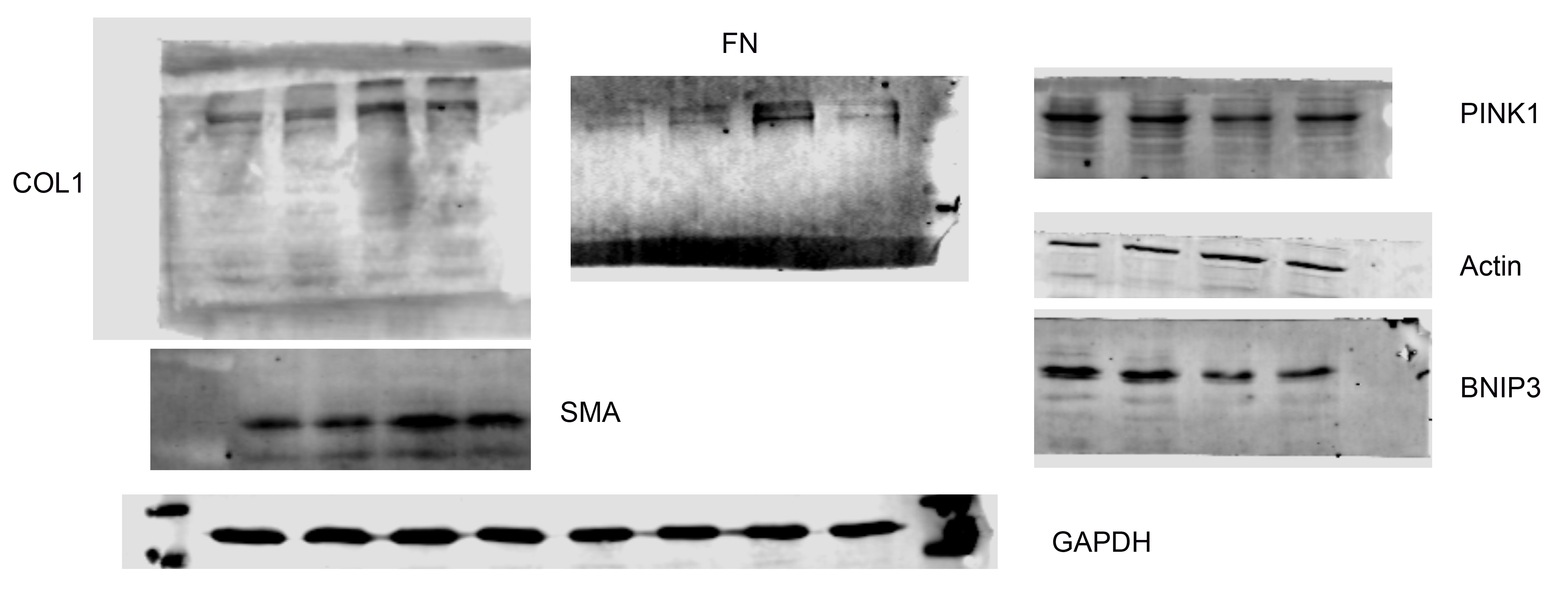

Supplement: Supplementary file 8 [file LSA-2023-02474_SdataF6.1.tif]

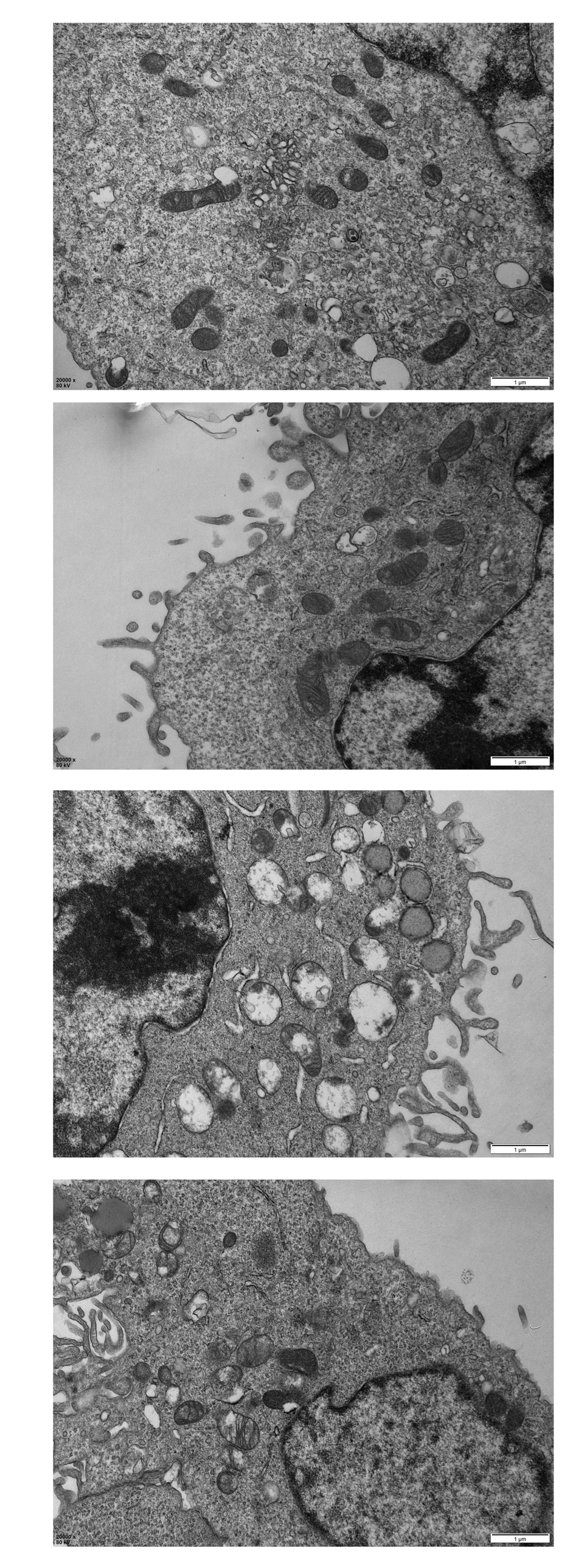

Supplement: Supplementary file 9 [file LSA-2023-02474_SdataF6.2.tif]

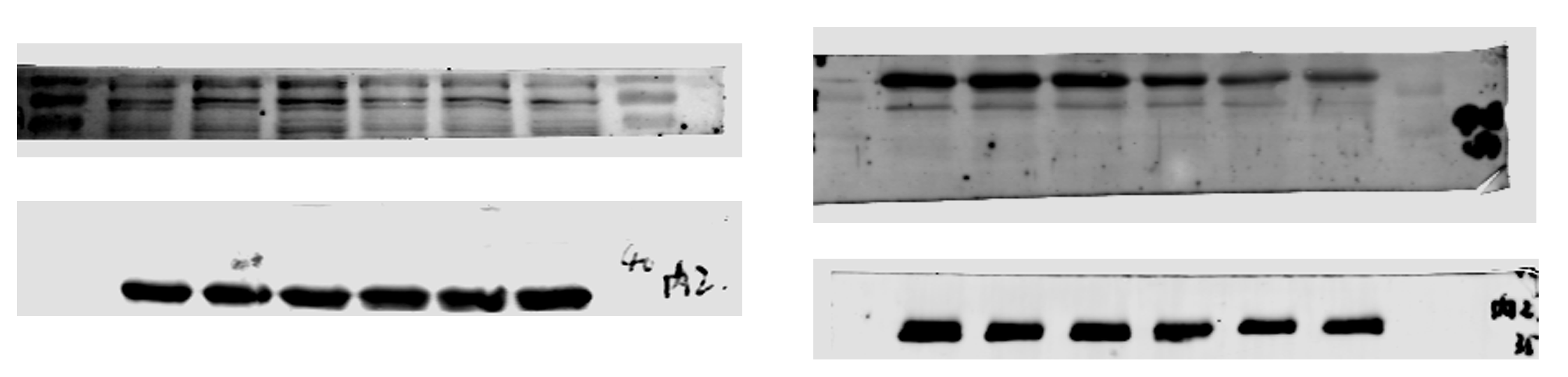

Supplement: Supplementary file 10 [file LSA-2023-02474_SdataF7.1.tif]

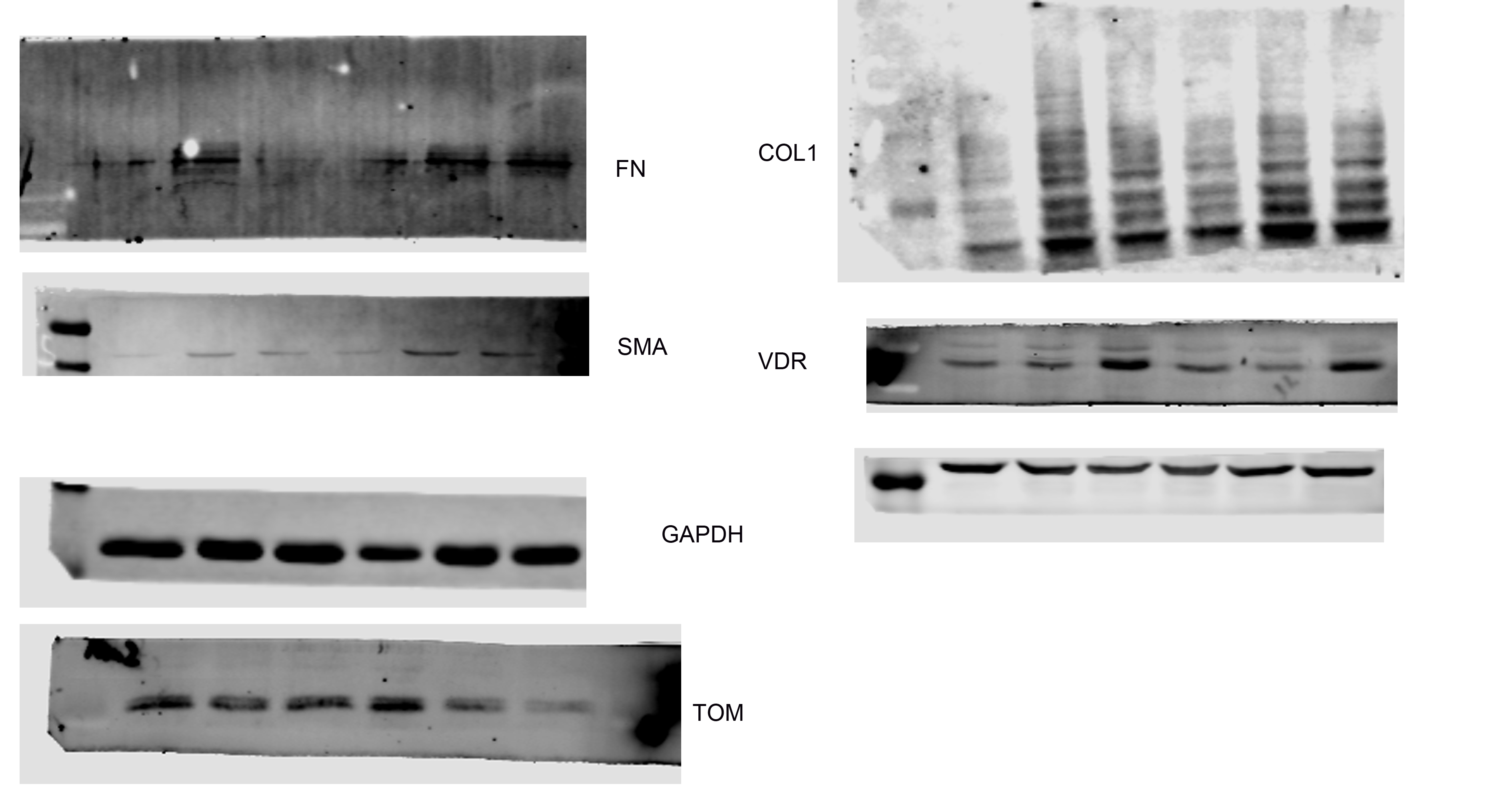

Supplement: Supplementary file 12 [file LSA-2023-02474_SdataF8.tif]
